# Hepatocyte growth factor activator inhibitor type-2 (HAI-2)/*SPINT2* contributes to invasive growth of oral squamous cell carcinoma cells

**DOI:** 10.18632/oncotarget.24450

**Published:** 2018-02-08

**Authors:** Koji Yamamoto, Makiko Kawaguchi, Takeshi Shimomura, Aya Izumi, Kazuomi Konari, Arata Honda, Chen-Yong Lin, Michael D. Johnson, Yoshihiro Yamashita, Tsuyoshi Fukushima, Hiroaki Kataoka

**Affiliations:** ^1^ Section of Oncopathology and Regenerative Biology, Department of Pathology, Faculty of Medicine, University of Miyazaki, Miyazaki, Japan; ^2^ Department of Oral and Maxillofacial Surgery, Faculty of Medicine, University of Miyazaki, Miyazaki, Japan; ^3^ Institute of Laboratory Animals, Kyoto University Graduate School of Medicine, Kyoto, Japan; ^4^ Lambardi Comprehensive Cancer Center, Georgetown University, School of Medicine, Washington, DC, USA

**Keywords:** oral squamous cell carcinoma, HAI-2, *SPINT2*, prostasin, HAI-1

## Abstract

Hepatocyte growth factor activator inhibitor (HAI)-1/*SPINT1* and HAI-2/*SPINT2* are membrane-anchored protease inhibitors having homologous Kunitz-type inhibitor domains. They regulate membrane-anchored serine proteases, such as matriptase and prostasin. Whereas HAI-1 suppresses the neoplastic progression of keratinocytes to invasive squamous cell carcinoma (SCC) through matriptase inhibition, the role of HAI-2 in keratinocytes is poorly understood. *In vitro* homozygous knockout of the *SPINT2* gene suppressed the proliferation of two oral SCC (OSCC) lines (SAS and HSC3) but not the growth of a non-tumorigenic keratinocyte line (HaCaT). Reversion of HAI-2 abrogated the growth suppression. Matrigel invasion of both OSCC lines was also suppressed by the loss of HAI-2. The levels of prostasin protein were markedly increased in HAI-2-deficient cells, and knockdown of prostasin alleviated the HAI-2 loss-induced suppression of OSCC cell invasion. Therefore, HAI-2 has a pro-invasive role in OSCC cells through suppression of prostasin. In surgically resected OSCC tissues, HAI-2 immunoreactivity increased along with neoplastic progression, showing intense immunoreactivities in invasive OSCC cells. In summary, HAI-2 is required for invasive growth of OSCC cells and may contribute to OSCC progression.

## INTRODUCTION

Hepatocyte growth factor activator inhibitor (HAI) is a type I transmembrane serine protease inhibitor. Thus far, two types of HAI have been identified: HAI-1 (encoded by the *SPINT1* gene) and HAI-2 (*SPINT2*), both of which have two extracellular Kunitz-type serine protease inhibitor domains, a transmembrane domain and a C-terminal short intracytoplasmic domain [1]. Whereas HAIs were initially identified as endogenous inhibitors of serum hepatocyte growth factor (HGF) activator, membrane-anchored serine proteases, such as a type II transmembrane serine protease (TTSP), matriptase and a glycosylphosphatidylinositol (GPI)-anchored protease, prostasin are primary targets of HAIs on the cell surface [2–4]. Therefore, HAIs likely have crucial roles in the regulation of pericellular activation of growth factors such as HGF and signaling mediated by protease-activated receptor 2 [2, 3]. It also appears that epithelial sodium channel activity is also regulated by HAIs, as prostasin and some TTSPs are involved in the activation of the epithelial sodium channel [5].

We have reported that the loss of HAI-1 in keratinocytes disturbs the normal keratinizing process and deranges the assembly of tonofilaments to desmosomes [6, 7]. In oral squamous cell carcinoma (OSCC), HAI-1 insufficiency results in enhanced invasion of OSCC cells and increased cancer-associated fibroblasts through dysregulated pericellular activity of matriptase [8, 9]. In fact, cell surface HAI-1 was decreased in invasive OSCC cells [8], and matriptase expression was increased in OSCC tissues predicting unfavorable prognosis of the patients [10]. However, the roles for HAI-2 in keratinocyte and squamous cell carcinoma (SCC), including OSCC, are poorly understood. Forced overexpression of HAI-1 or HAI-2 in keratinocytes has the potential to suppress the epidermal carcinogenesis and malignant progression in a matriptase transgenic mouse model using the keratin 5 promoter [11–13]. Therefore, both HAI-1 and HAI-2 have been implicated as suppressors in the neoplastic progression of keratinocytes. However, whereas HAI-1 is expressed in normal keratinocytes *in vivo*, the expression of HAI-2 in keratinocytes is hardly detectable in mice [14]. In contrast, immortalized human keratinocytes (HaCaT cells) and human SCC lines expressed notable levels of HAI-2 [15]. Therefore, endogenous HAI-2 may not function as a suppressor in neoplastic transformation of normal keratinocytes. Rather, it might be upregulated in transformed keratinocytes and contribute to the neoplastic progression.

OSCC is the sixth most common cancer worldwide, and mortality from OSCC is attributed to significant local invasiveness as well as regional and distant metastases [16, 17]. Therefore, the development of innovative treatment strategies requires a better understanding of the molecular mechanisms underlying the invasive growth of OSCC. In this report, using an immortalized human keratinocyte cell line (HaCaT) and two human OSCC cell lines, we analyzed premalignant and malignant keratinocytes for the roles of HAI-2 in cellular growth and invasion. Rather unexpectedly, HAI-2 loss significantly suppressed the growth and invasiveness of OSCC cells. In contrast, HAI-1 depletion enhanced invasiveness as reported previously [8], indicating the role of HAI-2 is distinct from that of HAI-1 in neoplastic keratinocytes. Immunohistochemical analysis of HAI-2 of surgically resected OSCC tissues, as well as expression analysis using a public database also suggested a positive role of HAI-2 in OSCC progression.

## RESULTS

### Generation of *SPINT2^−/−^* sublines from HaCaT, SAS and HSC3 cell lines

**Figure 1:**
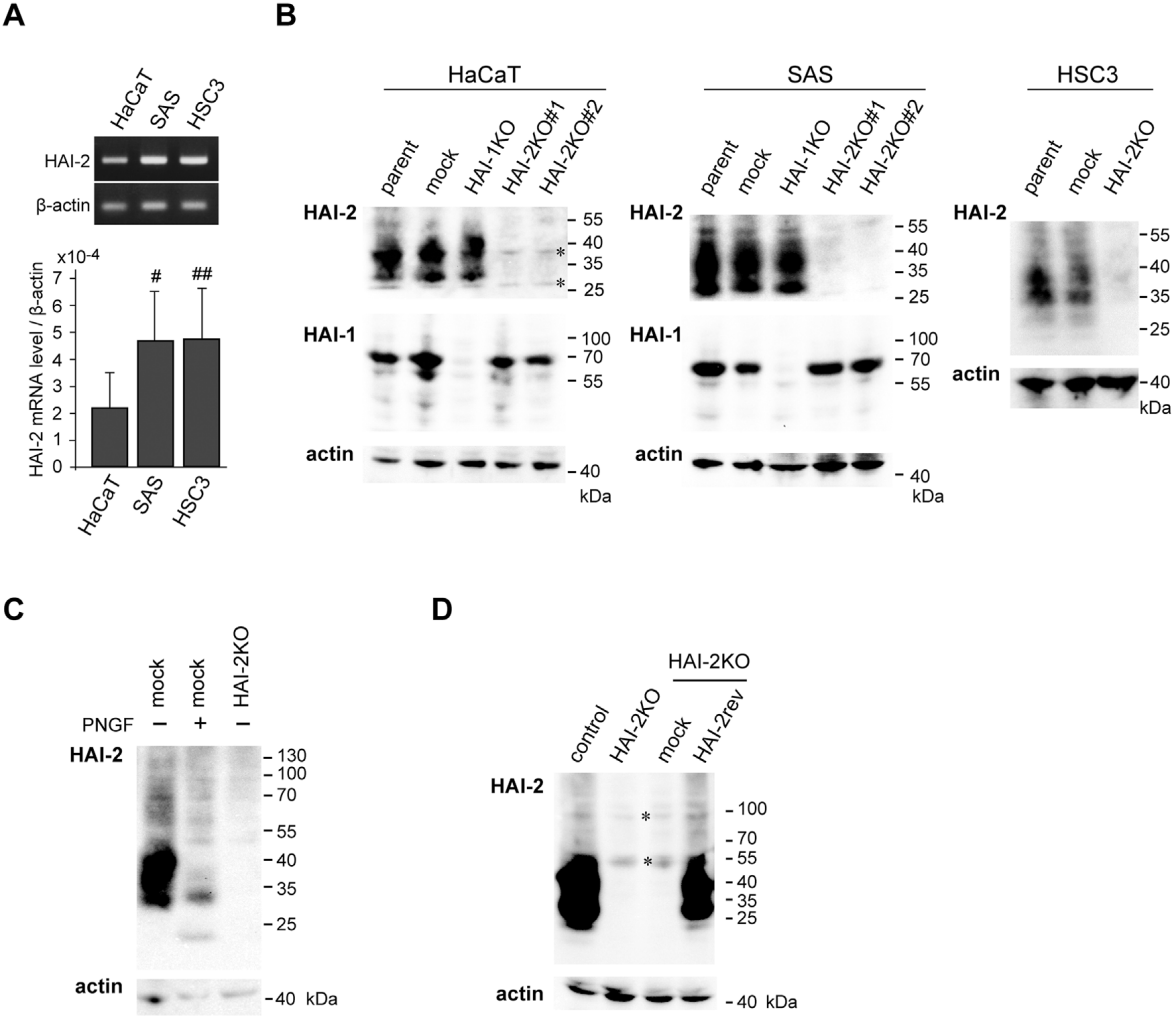
Expression of HAI-2 (*SPINT2*) in HaCaT and OSCC (SAS and HSC3) cell lines and the generation of *SPINT2* knockout sublines **(A)** A representative photo of reverse transcription polymerase chain reaction (RT-PCR) (upper panel) and semi-quantification of mRNA by quantitative RT-PCR (qRT-PCR) (lower panel). Data of qRT-PCR are mean ± standard deviation (SD) of four independent experiments. ^#^, *p* = 0.097; ^##^, *p* = 0.129, compared to HaCaT (Student’s t-test). **(B)** Generation of *SPINT2^−/−^* sublines (HAI-2KO^#^1 and ^#^2) and one *SPINT1^−/−^* sublines (HAI-1KO) in each of HaCaT or SAS cell line, as well as one SPINT2^−/−^ subline (HAI-2KO) in HSC3. Immunoblots for HAI-2 (mAb 2A6121) and HAI-1 (mAb M19) were performed using cellular extracts. β-actin was used as an internal loading control (actin). Specific HAI-2 bands in parent cells (parent) and mock-transfected cells (mock) were absent in HAI-2KO lines. ^*^, non-specific bands observed in all lanes. **(C)** Effects of PNGF treatment on HAI-2 of SAS cells. The same blot membrane was reprobed with β-actin antibody. **(D)** Reversion of HAI-2 in SAS/HAI-2KO#1 subline to generate SAS/HAI-2rev. Immunoblot for HAI-2 using extracts from control cells (control), SAS/HAI-2KO^#^1 cells (HAI-2KO), mock-transfected control cells from SAS/HAI-2KO#1 (mock) and SAS/HAI-2rev cells (HAI-2rev) is shown. ^*^, non-specific bands observed in all lanes. The same blot membrane was reprobed with β-actin antibody.

As it has been reported that HaCaT, SAS and HSC3 cell lines express HAI-2 protein, we initially compared the levels of mRNA for HAI-2. All three lines expressed HAI-2 (*SPINT2*) mRNA. Whereas the mRNA levels tended to be higher in OSCC lines than in HaCaT, the difference was not statistically significant (Figure 1A). To explore the role of HAI-2 in premalignant and malignant keratinocytes, we generated *SPINT2*^−/−^ sublines using a CRISPR/Cas9 system (Figure 1B). Two *SPINT2*^−/−^ sublines were successfully established from each HaCaT and SAS line (HaCaT/HAI-2KO#1, HaCaT/HAI-2KO#2, SAS/HAI-2KO#1 and SAS/HAI-2KO#2) and one *SPINT2*^−/−^ subline from HSC3 (HSC3/HAI-2KO) (Figure 1B). As these cell lines also express HAI-1 (Figure 1B; Supplementary Figure 1), one *SPINT1*^−/−^ subline was also generated from each HaCaT (HaCaT/HAI-1KO) or SAS (SAS/HAI-1KO) (Figure 1B). Genome sequencing confirmed a frameshift near the initiation codon of the *SPINT2* or *SPINT1* gene, followed shortly by an in-frame stop codon (Supplementary Figure 2). In all cell lines major HAI-2 proteins showed broad molecular weight (MW) bands around 30~45 kDa in SDS-PAGE under non-reducing condition. Treatment of the cellular extract with peptide N-glycosidase F (PNGF) revealed that the broad 30~45-kDa bands were N-glycosylated HAI-2 with complex glycosylation pattern (Figure 1C) [18]. We also generated a HAI-2 reversion cell line (SAS/HAI-2rev) by the transfection of the HAI-2 expression vector into SAS/HAI-2KO#1 (Figure 1D).

### The loss of HAI-2 suppressed growth of OSCC cells

**Figure 2:**
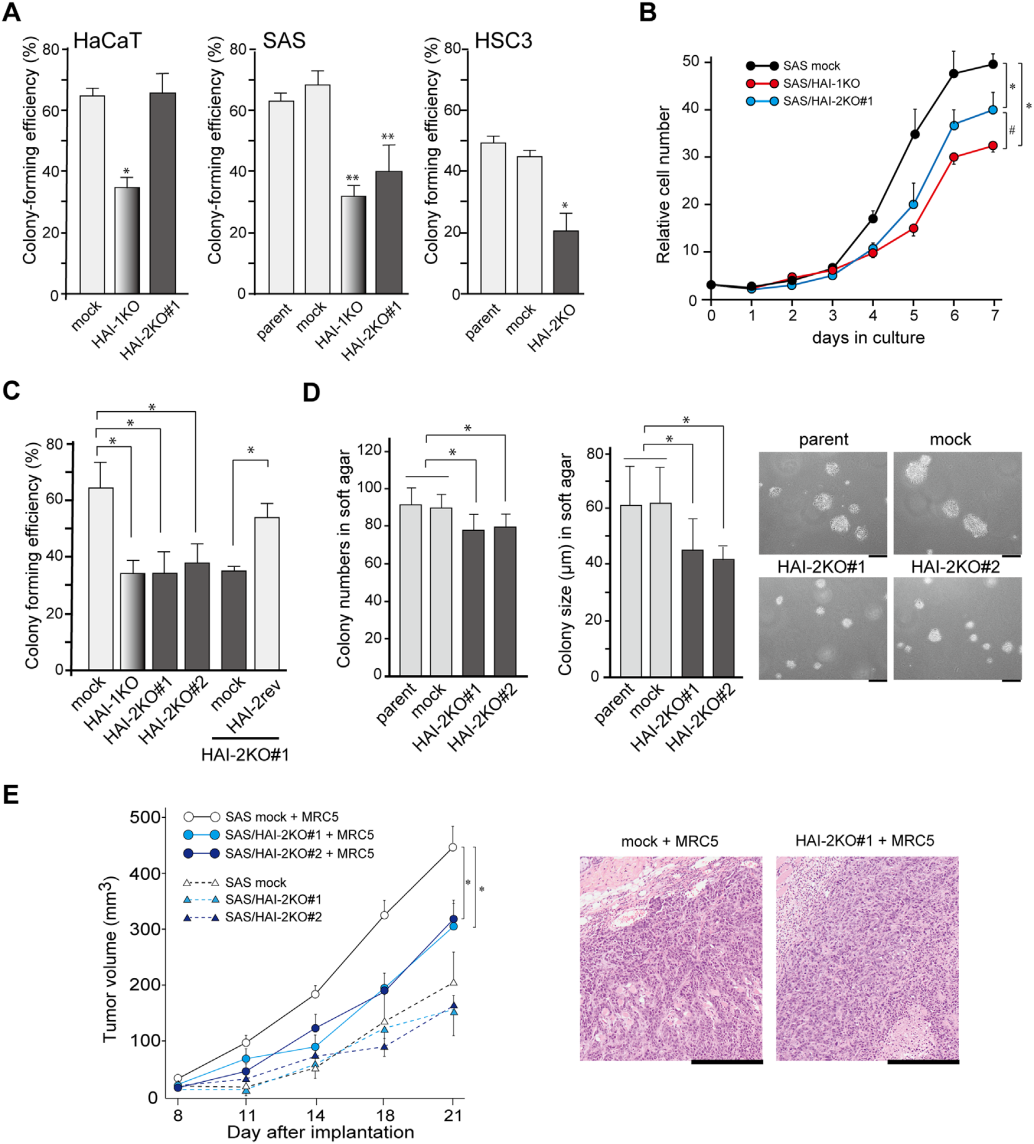
Effects of HAI-2-deficiency on growth properties *in vitro* in growth medium under normoxic condition and *in vivo* **(A)** Colony-forming efficiency of HaCaT, SAS and HSC3 cell lines and their mutant sublines. ^*^, *p* < 0.01 compared to mock and HAI-2KO^#^1 (HaCaT) or parent and mock (HSC3); ^**^, *p* < 0.001 compared to parent or mock; n = 6 in each group, Mann-Whitney U test. Error bars, SD. **(B)** Effects of HAI mutations on the growth curve of SAS cells. ^*^, *p* < 0.001; ^#^, *p* < 0.01; ANOVA with Fisher’s PLSD test. N = 3 in each group. Error bars, SD. **(C)** Effect of HAI-2 reversion on colony-forming efficiency of *SPINT2^−/−^* cells. ^*^, *p* < 0.05 Mann-Whitney U test; n = 6. Error bars, SD. **(D)** Effect of HAI-2-deficiency on anchorage-independent growth of SAS cells of in soft agar. Means ± SD of colony number per ×40 field (left graph) and colony diameter (right graph, μm) are indicated. N = 9 for each group; ^*^, *p* < 0.01 Mann-Whitney U test. Representative photos are also shown. Bar, 50 μm. **(E)** Effect of HAI-2 deficiency on *in vivo* tumor growth. Mock-transfected control SAS cells or SAS/HAI-2KO#1 were injected into the subcutaneous tissue of nude mice with or without MRC5 human fibroblasts. N = 5 for each group; ^*^, *p* < 0.0001 ANOVA with Fisher’s PLSD test. Error bars, standard error. Representative histology of formed SAS tumors transplanted with MRC5 fibroblasts is also shown (HE section; bar, 500 μm).

We analyzed the effect of HAI-2 deficiency on cellular proliferation *in vitro*. Whereas colony-forming efficiency of HaCaT cells was not altered by HAI-2 loss, SAS and HSC3 cells showed significantly reduced colony-forming efficiency on a culture dish in response to HAI-2 loss (Figure 2A). The same trend was observed in growth curve analysis (Figure 2B and Supplementary Figure 3). As reported previously [8], HAI-1-deficiency also suppressed the growth of SAS cells, and in contrast to HAI-2, HaCaT cells also showed suppressed growth in the absence of HAI-1 (Figure 2A and Supplementary Figure 3). Reversion of HAI-2 alleviated the suppression of colony formation (Figure 2C). Anchorage-independent growth in soft agar was also suppressed by HAI-2 loss in SAS cells (Figure 2D). Then, we analyzed the effect of *SPINT2* deletion on tumor formation in nude mice using the SAS sublines. We used two implantation methods for this study. One was transplantation of SAS cells only. Another method was transplantation of a mixture of SAS cells and MRC5 human fibroblasts. The mean size of *in vivo* tumors was significantly larger when MRC5 cells were concomitantly transplanted (Figure 2E). In agreement with the results of the *in vitro* growth study, *SPINT2*^−/−^ cells formed smaller tumors than control SAS cells, but notable differences in histology were not observed (Figure 2E). Metastasis was not observed in either group after the observation period (3 weeks).

### HAI-2 was required for *in vitro* invasion of OSCC cells

**Figure 3:**
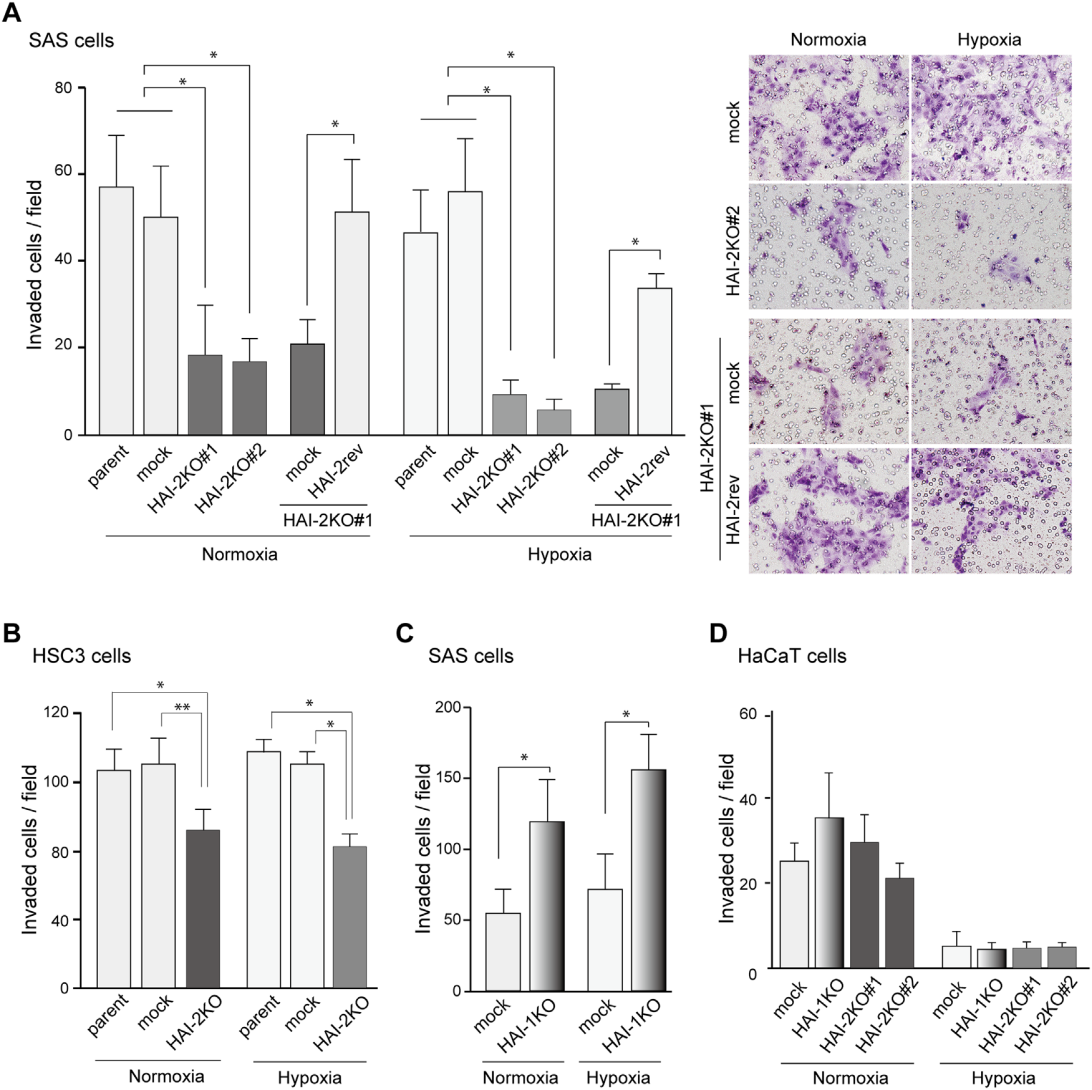
Effect of HAI-2-deficiency on Matrigel invasion of OSCC cells **(A)** Decreased Matrigel invasiveness by SAS cells after *SPINT2* deletion and effect of HAI-2 reversion. Data of both normoxic and hypoxic conditions are shown. ^*^, *p* < 0.001 Mann-Whitney U test; n = 12; error bar, SD. **(B)** Decreased Matrigel invasiveness by HSC3 cells after *SPINT2* deletion. ^*^, *p* < 0.01; ^**^, *p* < 0.05 Mann-Whitney U test; n = 8; error bar, SD. **(C)** Increased Matrigel invasiveness after *SPINT1* deletion from SAS cells. ^*^, *p* < 0.0001 Mann-Whitney U test; n = 24; error bar, SD. **(D)** Effects of HAI mutations on Matrigel invasiveness by HaCaT cells.

Previously, we reported that silencing of *SPINT1* by short hairpin RNA enhanced the invasive capacity of cancer cells, including OSCC cells [8, 19–21], indicating that HAI-1 is a suppressor of cancer cell invasion. Therefore, we asked whether HAI-2 also suppressed cellular invasion of OSCC cells. The invasion front of cancer is frequently hypoxic. Therefore, we analyzed the cells’ invasive capability under both normoxic and hypoxic conditions. Unexpectedly, the loss of HAI-2 significantly suppressed the invasion of SAS cells under both normoxic and hypoxic conditions. Moreover, reversion of HAI-2 in *SPINT2*^−/−^ cells eliminated the suppression of invasion induced by loss of HAI-2 (Figure 3A). The pro-invasive role of HAI-2 was also observed in another OSCC cell line, HSC3 (Figure 3B). It is unlikely that the suppression of invasion was caused by the reduced growth rate in the absence of HAI-2 because the invasion assay was performed within 48 h of plating and no significant difference of cellular growth was observed in this assay period as shown in Figure 2B and Supplementary Figure 3. In contrast to *SPINT2*, knockout of *SPINT1* resulted in enhanced Matrigel invasion capacity of SAS cells, confirming the previously reported anti-invasive role of HAI-1 in OSCC [8] (Figure 3C). On the other hand, the invasive capability of HaCaT was lower compared to the SAS and HSC3 OSCC cell lines. In HaCaT cells, *SPINT2* knockout did not suppress the invasion significantly (Figure 3D). Therefore, HAI-2 was required for invasion of OSCC cells, but not that of HaCaT premalignant keratinocytes. Taken together, this loss-of-function study revealed that the effects of HAI-2 on the invasive capacity of OSCC cells are quite different from those of HAI-1. Whereas the loss of HAI-1 enhanced cellular invasion but suppressed proliferation, loss of HAI-2 suppressed both invasion and proliferation.

### Enhanced expression of prostasin in response to *SPINT2* deletion

**Figure 4:**
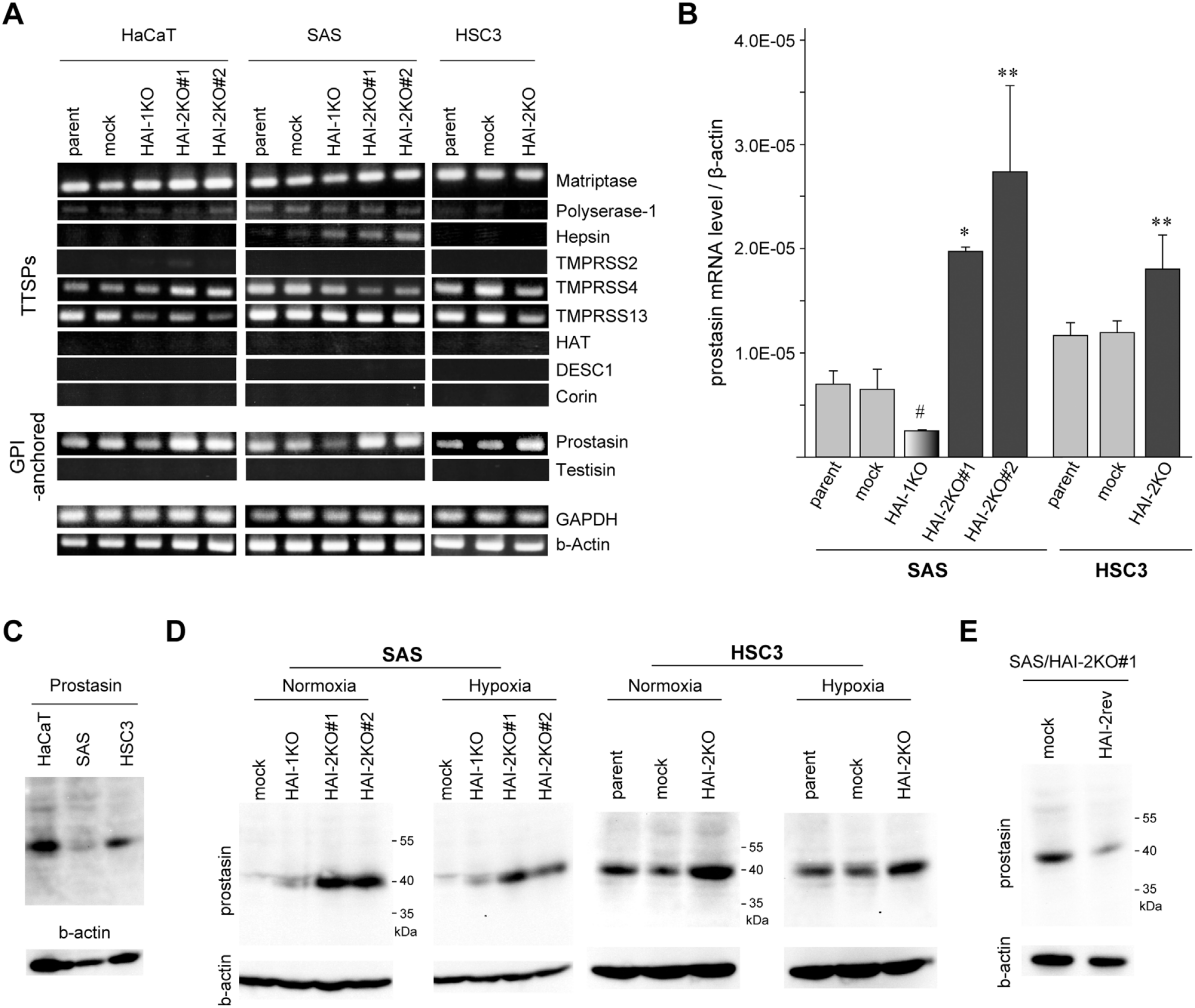
Loss of HAI-2 upregulated prostasin **(A)** RT-PCR analyses of nine TTSPs and two GPI-anchored serine proteases in HaCaT, SAS, HSC3 and their mutants. **(B)** Quantitative RT-PCR for prostasin mRNA in SAS, HSC3 and their mutants. ^*^, *p* < 0.001 compared to parent and mock; ^**^, *p* < 0.05 compared to parent and mock; ^#^, *p* < 0.05 compared to parent; Student’s t-test, n = 3 for each group. **(C)** Immunoblot analysis of prostasin. Prostasin proteins in cellular extracts from parental lines of HaCaT, SAS and HSC3 were compared. **(D)** Immunoblot analysis of prostasin in extracts of SAS, HSC3 and their mutant lines cultured under normoxic or hypoxic conditions. **(E)** Effect of HAI-2 reversion (HAI-2rev) in SAS/HAI-2KO#1 cells on the prostasin protein level.

Next, we analyzed the molecular mechanism by which *SPINT2* deletion suppressed OSCC cell migration and invasion. Gelatin zymography of serum-free culture supernatant showed gelatinolytic bands sensitive to GM6001, suggesting the presence of pro-matrix metalloprotease (MMP)-2 and -9; however, significant alterations of the gelatinolytic bands were not observed by the deletion of *SPINT2* (Supplementary Figure 4A). We also examined the expression of E-cadherin and vimentin to test a possibility that HAI-2 loss induced epithelial to mesenchymal transition (EMT), but the expression of these molecules was not altered, either (Supplementary Figure 4B). Recent evidence suggests that the most important target protease for HAI-2 in the epithelial cell is prostasin, which is likely essential for the regulated activity and localization of matriptase as well as for epithelial integrity [4, 22, 23]. Other membrane-anchored serine proteases, particularly TTSPs, may also be targets of HAI-2 [24]. Therefore, we compared control cells and HAI-2 mutants for the expression levels of prostasin, matriptase and other membrane-anchored serine proteases. In the initial screening by reverse transcription-polymerase chain reaction (RT-PCR), proteases that showed notable expression in all cell lines included matriptase, TMPRSS4, TMPRSS13 and prostasin (Figure 4A). Among them, the prostasin mRNA level was consistently increased in all *SPINT2*-deleted sublines. The enhanced prostasin expression in *SPINT2*-deleted OSCC cells was further confirmed by quantitative RT-PCR analysis (Figure 4B). We also analyzed the cellular prostasin protein levels. The endogenous prostasin protein levels were lower in OSCC cells than in HaCaT cells (Figure 4C), and loss of HAI-2 also upregulated the prostasin protein levels in both SAS and HSC3 cells (Figure 4D). Consequently, the reversion of HAI-2 in *SPINT2*^−/−^ SAS cells downregulated the prostasin level (Figure 4E).

### Prostasin upregulation was responsible for *SPINT2*-deletion-mediated suppression of cellular invasiveness

**Figure 5:**
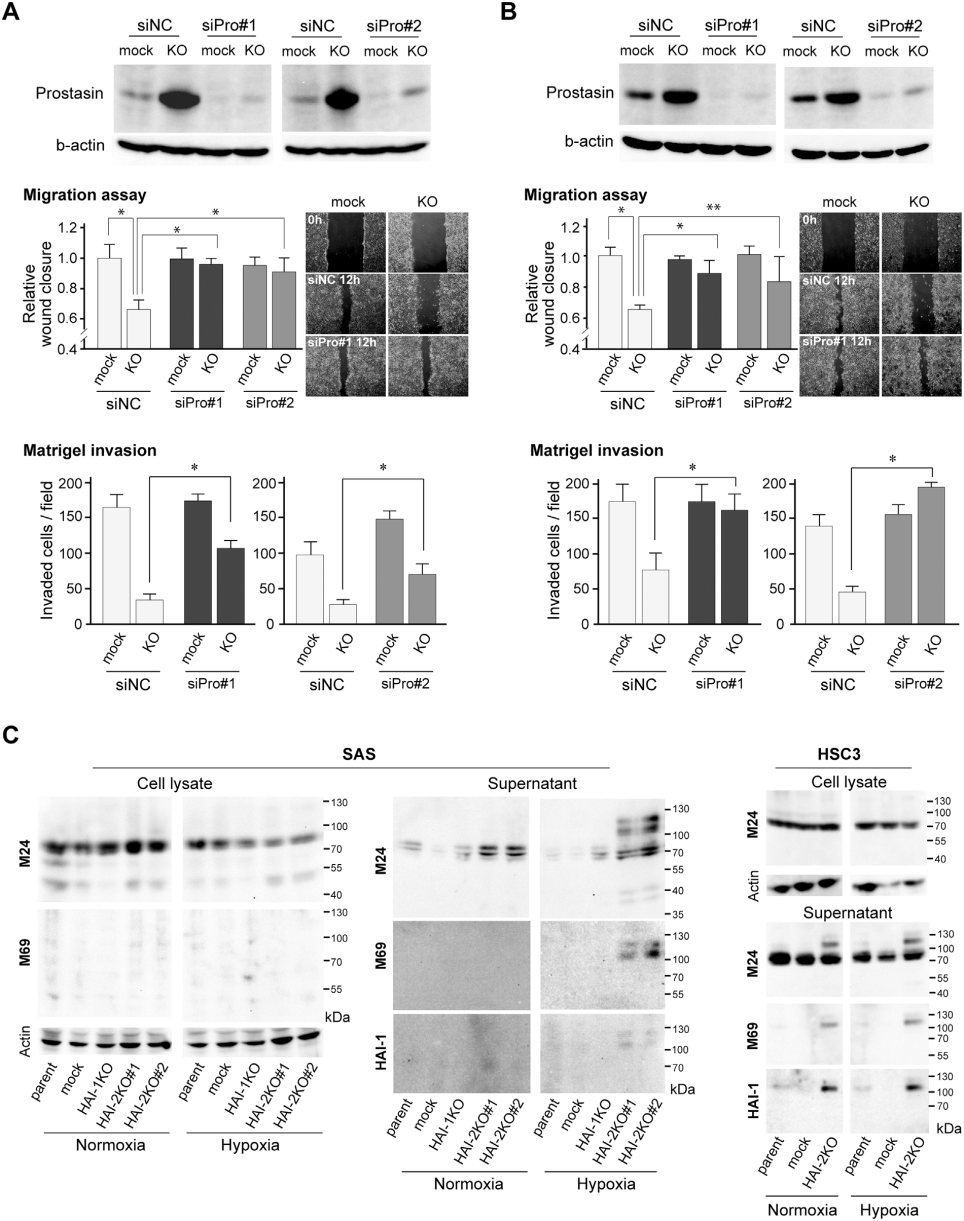
Effects of prostasin silencing on HAI-2 loss-mediated suppression of invasiveness **(A, B)** Effect of prostasin silencing by prostasin siRNA^#^1 (siPro^#^1) and ^#^2 (siPro^#^2) on migration in wound healing assays and Matrigel invasiveness by *SPINT2^−/−^* SAS (SAS/HAI-2KO^#^1) (A) and *SPINT2^−/−^* HSC3 cells (B) under normoxic condition. Two kinds of prostasin siRNAs were used and the extents of silencing are shown in the top panel. ^*^, *p* < 0.01; ^**^, *p* < 0.05; n = 8, Mann-Whitney U test. Photos are represented images of mock-transfected control and HAI-2KO cells 0 h (control siRNA) and 12 h (control siRNA and prostasin siRNA^#^1) after wounding. All images for each set wound healing assay experiments are presented in Supplementary Figure 5A. **(C)** Effects of HAI mutations on matriptase activation and shedding in OSCC cell lines. Data under normoxic and hypoxic conditions are shown. Anti-total matriptase mAb (M24) and anti-activated matriptase mAb (M69) were used for detection of matriptase. For supernatants, the same blot was reproved by anti-HAI-1 mAb M19.

Next, we asked whether increased prostasin was responsible for the reduced invasiveness of OSCC cells due to the loss of HAI-2. To address this question, we examined how siRNA-mediated silencing of prostasin affected the migration and invasion of *SPINT2*-deleted OSCC cells. As shown in Figure 5A (SAS cells), Figure 5B (HSC3 cells) and Supplementary Figure 5, the silencing of prostasin resulted in the recovery of migratory and invasive capabilities of *SPINT2*^−/−^ OSCC cells as judged by wound healing assays and Matrigel invasion assays, respectively, under normoxic conditions. Similar results were obtained under hypoxic condition (Supplementary Figure 6). In contrast, the silencing of prostasin did not affect the motility of control (*SPINT2*^+/+^) cells. Therefore, endogenous HAI-2 may be sufficient to suppress the anti-motility function of prostasin. Decreased colony sizes in soft agar observed in HAI-2KO SAS was also alleviated by the silencing of prostasin, though the colony number was not altered (Supplementary Figure 5B). On the other hand, cellular growth rate on culture dish was not recovered by the silencing of prostasin (Supplementary Figure 6), indicating the prostasin upregulation was involved in the decreased invasiveness and anchorage-independent growth but not in the decreased growth rate on culture dishes observed after *SPINT2* deletion.

Recent studies indicated that prostasin mediates matriptase activation both enzymatically and nonenzymatically [25], whereas this prostasin function in matriptase zymogen activation is limited to those cells with exogenous expression of matriptase and prostasin [26]. Thus, increased prostasin may contribute to matriptase activation. Therefore, we examined matriptase activation using antibodies that recognized total matriptase (M24) and activated matriptase (M69). Notably, activated matriptase was increased in the absence of HAI-2 in OSCC cells. However, the activated matriptase was detectable mostly in the culture supernatant, which was complexed with HAI-1 (Figure 5C). Therefore, whereas the excess activation of matriptase occurred in the absence of HAI-2, it was inhibited by HAI-1.

### Increased HAI-2 expression along with neoplastic progression of the oral epithelium

**Figure 6:**
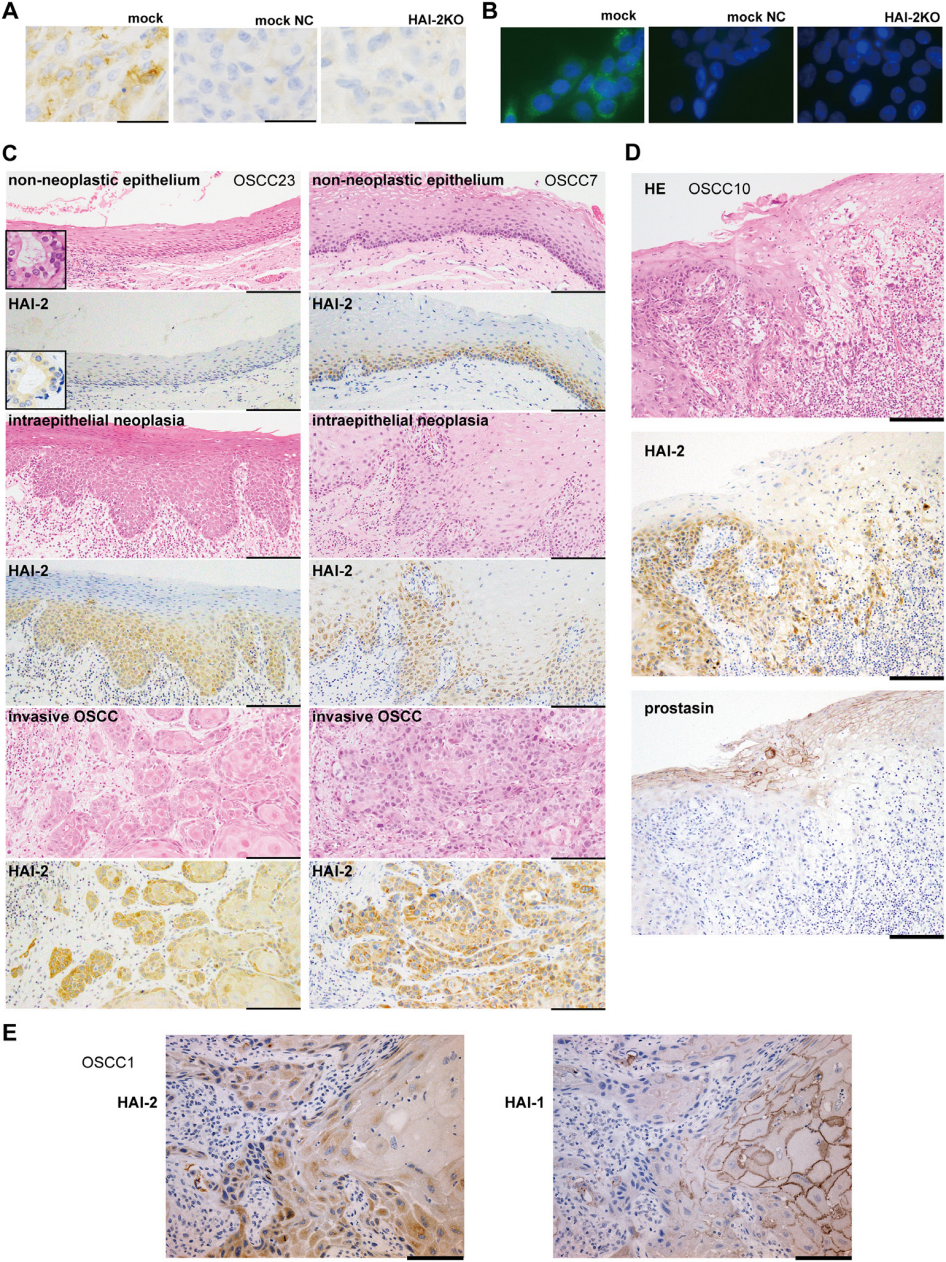
Immunohistochemistry of HAI-2 in OSCC tissues **(A)** Validation of specificity of the primary antibody (XY9) by immunohistochemistry. Xenotransplanted tumors of control SAS (mock) and SAS/HAI-2KO^#^1 (HAI-2KO) were immunostained with XY9 mouse mAb against human HAI-2. Intracytoplasmic HAI-2 immunoreactivity was noted in the control cells. Faint immunoreactivity was observed by omitting the primary antibody (mock NC), suggesting a reaction of the secondary antibody to mouse IgG. Immunostaining of HAI-2KO tumor resulted in a pattern similar to mock NC. **(B)** Validation of the XY9 mAb by immunocytochemistry. No immunoreactivity was observed in SPINT2*^−/−^* (HAI-2KO) SAS cells. **(C)** Immunohistochemistry of surgically resected human OSCC tissues by XY9 mAb. Representative results of non-neoplastic stratified epithelium, intraepithelial neoplasia and invasive OSCC in the same specimen from OSCC case No.23 (OSCC23, left column) and OSCC7 (right column). Note that whereas non-neoplastic epithelium shows faint or distinct immunoreactivity depending on the case, neoplastic lesions are consistently positive, indicating higher expression levels compared to the non-neoplastic epithelium. Insets indicate internal positive control (minor salivary gland epithelium). Bars, 200 μm. **(D)** Comparative immunohistochemistry of HAI-2 and prostasin in serial sections of OSCC and adjacent non-malignant epithelium (OSCC10). Note that HAI-2 is strongly expressed in the overt cancer cells, whereas prostasin is preferentially expressed in differentiated keratinocytes, showing a reciprocal expression pattern. Bar, 200 μm. **(E)** Comparative immunohistochemistry of HAI-1 (mAb 1N7) and HAI-2 in serial sections of invasive OSCC (OSCC1). The cancer cells at the invasive front are mostly negative for membranous HAI-1, whereas they are strongly positive for HAI-2. Bar, 100 μm.

The above *in vitro* and *in vivo* observations indicated that the function of HAI-2 was distinct from that of HAI-1 in neoplastic keratinocytes, and unlike HAI-1, HAI-2 expression may contribute to OSCC progression. To test the *in vivo* relevance of the findings in human subjects, we analyzed the expression of HAI-2 in normal, premalignant and cancerous oral mucosa using surgically resected tissues obtained from OSCC patients. Formalin-fixed paraffin-embedded tissue sections from 25 OSCC cases were subjected to an immunohistochemical study of HAI-2. Before immunostaining, the coexistence of portions of non-neoplastic oral epithelium, intraepithelial neoplasia and invasive OSCC in a single tissue section was confirmed with hematoxylin-eosin (HE) stain. The specificity of the primary antibody was verified by the absence of immunoreactivity of *SPINT2*^−/−^ SAS cells (Figures 6A, 6B). In the non-neoplastic epithelium, HAI-2-high (i.e., score ≥ 3) immunoreactivity was observed in 36% (9/25) of the cases showing positive reaction predominantly in the parabasal cells and 16 cases showed negative or borderline immunoreactivity (i.e., HAI-2-low; score ≤ 2) (Table 1, Figure 6C). The inconsistent immunoreactivity in the non-neoplastic epithelium may be due to decreased antigen preservation during storage of the paraffin block. However, the HAI-2-high immunoreactivity was consistently observed in the portions of dysplasia or carcinoma *in situ* in the same section (92%, 23/25) (Table 1, Figure 6C). In the invasive OSCC portion, 96% of the cases (24/25) showed HAI-2-high immunoreactivity, and 14 cases (56%) showed intense immunoreactivity in more than 50% of the cancer cells (i.e., score 4) (Table 1, Figure 6C). The difference of HAI-2-high rate between non-neoplastic epithelium and intraepithelial neoplasia or non-neoplastic epithelium and invasive carcinoma was statistically significant (*p* < 0.0001, Fisher’s exact test). We also immunostained HAI-2 and prostasin in serial sections from representative four OSCC cases. Prostasin showed a reciprocal immunostaining pattern to HAI-2 in OSCC and adjacent oral epithelium (Figure 6D). Of note, the HAI-2 immunoreactivity was present in the cytoplasm. This subcellular localization pattern is compatible with previous studies of other epithelial and cancer cells [15, 27, 28]. In contrast, HAI-1 showed a clear membrane-associated pattern with reduced immunoreactivity at the invasive front of OSCC as reported [8] (Figure 6E).

**Table 1:** HAI-2 immunoreactivity score of non-neoplastic oral epithelium, intraepithelial neoplasia and invasive OSCC

	HAI-2-low cases			HAI-2-high cases	
	score 0	score 1	score 2	score 3	score 4
Non-neoplastic epithelium	11	4	1	7	2
Intraepithelial neoplasia	1	1	0	15	8
Invasive OSCC	0	0	1	10	14

Because the number of cases subjected to the immunohistochemical study was very limited (25 cases) and most OSCC cases were positive for HAI-2, we could not perform conclusive evaluation for the relationship between HAI-2 immunoreactivity to clinicopathological parameters. Therefore, to further analyze whether HAI-2 was in fact expressed in OSCC and had prognostic impact on the patients suffered from OSCC, we performed gene expression analysis using RNA sequence data in the cancer genome atlas (TCGA) public database. Notable levels of *SPINT2* mRNA were confirmed in OSCC, showing the median value a little lower than pancreatic ductal adenocarcinomas that are known to overexpress *SPINT2* [29] (Supplementary Figure 7A). On the other hand, some cancers known to show *SPINT2* promoter hypermethylation [30–32] showed very low levels of *SPINT2* mRNA. Then, using 377 cases of OSCC in the TCGA database, we analyzed the prognostic significance of markedly high *SPINT2* expression (≥ mean + 0.5 SD; n = 49). However, we could not detect statistically significant relationship between high *SPINT2* expression and shorter overall survival (OS) (*p* = 0.068) (Supplementary Figure 7B).

## DISCUSSION

Pericellular proteolysis plays a crucial role in shaping the tumor microenvironment by modulating the extracellular matrix and processing numerous bioactive molecules, thereby contributing to both malignant and nonmalignant phenotypes [33, 34]. In our previous studies, we reported that loss of cell surface HAI-1 contributed to invasion of OSCC cells through dysregulation of its targeted protease, matriptase [8, 9], but suppressed *in vitro* cellular proliferation [8]. In this study, depletion of HAI-2 also suppressed cell growth; however, in contrast to loss of HAI-1, the loss of HAI-2 significantly suppressed the migration and invasion of OSCC cells *in vitro*. Consequently, we observed strong HAI-2 immunoreactivity in invasive cancer cells of resected OSCC tissues. The high expression of *SPINT2* mRNA in OSCC was also confirmed by the TCGA public database.

The precise function of HAI-2 *in vivo* is unclear. However, there is evidence for a physiological role for HAI-2 based on the discovery of *SPINT2* mutations in the syndromic form of congenital sodium diarrhea [35]. In intestinal epithelial cells, HAI-2 likely regulates prostasin [22, 36]. The established critical roles of HAI-2 in embryogenesis and placenta formation are based on the studies of mutant mice. Similar to the intestinal epithelium, the observed phenotypes in *Spint2^−/−^* mice involve dysregulation of the prostasin-matriptase axis [4]; however, apparent phenotypes were not reported in the skin and oral mucosa. In the murine skin and oral mucosa, keratinocytes did not express a notable level of HAI-2 *in vivo* when analyzed in beta-galactosidase-tagged *Spint2* mice [14]. The current immunohistochemical study showed that HAI-2 was expressed in the normal oral epithelium in human and the immunoreactivity was upregulated in OSCC and its precursor lesions. Taken altogether, the high expression of HAI-2 in OSCC is likely an acquired characteristic along with malignant transformation.

The precise molecular mechanism by which loss of HAI-2 suppressed the invasiveness and growth of OSCC cells remains to be determined. Our current experimental evidence suggested that the pro-invasive function of HAI-2 may be mediated, at least partly, by downregulation of prostasin function. The prostasin downregulation may also contribute partly to HAI-2-mediated enhancement of the anchorage-independent growth in soft agar. Indeed, an anti-invasive role of prostasin has been reported in various tumors. Prostasin inhibited invasion of prostatic cancer cells [37], breast cancer cells [38] and choriocarcinoma cells [39]. Loss of prostasin induced metastasis of hepatocellular carcinoma cells [40] and enhanced EMT of urothelial carcinoma cells [41]. Regarding squamous cell carcinomas, reduced expression of prostasin was recently reported in esophageal squamous cell carcinoma, and forced expression of prostasin suppressed the invasive growth of the cells *in vitro* [42]. Therefore, prostasin is a suppressor of the malignant phenotype in a wide range of cancers, explaining why reduced expression of prostasin in cancer cells correlated with less differentiated histology and poorer prognosis of the patients [40, 42–45]. In the current study, it remains to be determined how prostasin suppressed the invasiveness of OSCC cells. EMT is unlikely, as the E-cadherin level was not altered by HAI-2 loss. Whereas we observed increased cellular levels of prostasin protein and mRNA in response to HAI-2 loss, according to the molecular size detected by immunoblotting, the increased prostasin may be a zymogen form. Nonetheless, protease activity-independent functions of prostasin have been reported. The inactive prostasin somehow functions in embryonic and postnatal developmental process [4]. Moreover, inactive prostasin downregulates the expression of molecules involved in malignant phenotypes of cancer cells, such as urokinase-type plasminogen activator (uPA), uPA receptor, and cyclooxygenase 2 [46]. Further mechanistic analysis for the prostasin function in OSCC cells will be required in future studies. On the other hand, prostasin was not involved in the suppression of cellular growth rate on culture dishes caused by *SPINT2* deletion in this study, and the mechanism by which HAI-2 contributes to cellular proliferation is currently unknown. As either HAI-1-deficiency or HAI-2-deficiency suppressed the *in vitro* growth of OSCC cells, tight regulation of membrane-anchored protease activities may be important for cellular proliferation.

In contrast with the present data, many studies (including our own) have indicated that HAI-2 has a tumor-suppressor function. Indeed, significant downregulation of HAI-2 has been observed in malignant brain tumors, hepatocellular carcinomas, renal cell carcinomas and melanomas [30–32, 47–50]. Moreover, the TCGA database also indicated significantly reduced *SPINT2* mRNA levels in these cancer types. In HAI-2-low types of cancer, downregulation of HAI-2 was mediated by promoter hypermethylation of the *SPINT2* gene [30–32], and recently, *SPINT2* hypermethylation was reported in other cancer types, such as esophageal carcinoma and gastric cancer [51, 52]. In prostatic cancer, decreased HAI-2 level was observed along with cancer progression [53, 54]. Consequently, forced overexpression of HAI-2 in cancer cells with reduced HAI-2 expression suppressed their capabilities for invasive growth [47, 49, 54–56]. On the other hand, the *SPINT2* gene was initially identified as a gene overexpressed in pancreatic cancer under the name of *Kop* (Kunitz domain containing protein overexpressed in pancreatic cancer) [29], and HAI-2 protein was initially discovered in and purified from culture supernatant of a gastric carcinoma cell line [2]. Indeed, the TCGA database indicates high *SPINT2* mRNA levels in pancreatic ductal adenocarcinomas and the median level of *SPINT2* mRNA is high in other major cancers such as non-small cell lung cancers, colon cancers, breast cancers and prostatic cancers. In breast cancer, HAI-2 expression was high in *HER2*-positive tumors, which was further induced by hypoxia [57]. Moreover, high HAI-2 expression in breast cancer was a significant predictor for poor clinical treatment response rate [57]. In OSCC, our current study showed that the expression of HAI-2 increased during disease progression. Oral intraepithelial neoplasia/dysplasia showed increased immunoreactivity compared to the adjacent normal epithelium, and most invasive carcinoma cases showed intense immunoreactivity at least some of the cancer cells.

Based on the foregoing, we suggest that the role of HAI-2 is cell type- or tissue type-specific, possibly depending on membrane-anchored proteases. An alternative explanation for the discrepancy between our current study and previous reports could be due to the different approaches used to analyze HAI-2 function. For example, previous studies applied a forced overexpression strategy to cancer cells with reduced HAI-2 expression. In contrast, our current report utilized a loss-of-function analysis by homozygous knockout of the *SPINT2* gene in cancer cells with sufficient HAI-2 expression. Another question unanswered in this study is the mechanism by which HAI-2 deficiency induces prostasin upregulation. Clearly, further studies to clarify the link between HAI-2 and prostasin expression will be required.

In conclusion, this study suggests, for the first time, that HAI-2 contributes to the invasive growth of OSCC cells. Our study also provides evidence that HAI-2 may serve as a histopathological marker of neoplastic progression of oral epithelium. Further studies to clarify the molecular interactions underlying the pro-invasive function of HAI-2 will shed light on the novel mechanism regulating the invasive growth of OSCC, helping the development of innovative therapies.

## MATERIALS AND METHODS

### Antibodies

The following antibodies were used: anti-human HAI-1 goat polyclonal antibody (R&D Systems, Minneapolis, MN, USA), anti-human prostasin mouse mAb (BD Biosciences, Franklin Lakes, NJ), anti β-actin mouse mAb (Sigma, St Louis, USA), anti-human HAI-1 mouse monoclonal antibody (mAb) M19 and 1N7, and anti-human HAI-2 mouse mAbs XY9 and 2A6121, anti-human matriptase (total matriptase) mouse mAb M24 and anti-human activated matriptase mouse mAb M69. Preparation and characterization of the mAbs M19, XY9, M24, M69 and 1N7 have been reported previously [18, 58–60]. Another anti-human HAI-2 mouse mAb 2A6121, raised against the presumed extracellular portion of recombinant HAI-2, was prepared in our laboratory.

### Cell culture

A spontaneously transformed immortal keratinocyte line, HaCaT, was obtained from the CLS Cell Lines Service GmbH (Eppelheim, Germany). Two human OSCC cell lines, SAS and HSC3, were obtained from the Cell Resource Center for Biomedical Research, Tohoku University (Sendai, Japan) and the Riken Cell Bank (Tsukuba, Japan), respectively. A human fibroblasts cell line, MRC5, was obtained from the Japanese Cancer Research Resources Bank (Tokyo, Japan). These cells were cultured in Dulbecco’s modified Eagle’s medium (DMEM) containing 10% fetal bovine serum (FBS), with penicillin G and streptomycin. All cultured cells were maintained at 37°C in a humidified incubator with 5% CO_2_ (normoxic culture). For hypoxic cultivation, cultured cells were maintained at 37°C in a humidified incubator with 5% CO_2_, 1% O_2_ and 94% N_2_ using a CO_2_ multi-gas incubator AP30 (ASTEC, Fukuoka, Japan). Serum-free culture was performed in DMEM supplemented with 1.72 μM insulin, 69 nM transferrin and 39 nM of sodium selenite (ITS) (Thermo-Fisher, Waltham, MA, USA).

### Knockout (KO) of the *SPINT1* or *SPINT2* gene in cultured cells

Genome editing using clustered regularly interspaced short palindromic repeat (CRISPR)/ CRISPR-associated proteins 9 (Cas9) was used for KO of the *SPINT1* or *SPINT2* gene in the HaCaT, SAS or HSC3 cell line. A Cas9 and single guide RNA (sgRNA) expression plasmid, pSpCas9(BB)-2A-Puro(px459), was purchased from ADDgene (Cambridge, MA, USA). To generate sgRNA for Cas9 targets, we designed two 20-nt target sequences preceding a 5′-NGG of a protospacer adjacent motif (PAM) sequence. The sgRNA target sequences of *SPINT1* (HAI-1) and *SPINT2* (HAI-2) were as follows: *SPINT1* forward, 5′-CACCGGAAGGCGATGGCCCCTGCG-3′ and reverse, 5′-AAACCGCAGGGGCCATCGCC TTCC-3′; *SPINT2* forward, 5′-CACCGTGCGGGCT GAGGCGGAGCC-3′ and reverse, 5′-AAA CGGCTCCGCCTCAGCCCGCAC-3′. These single-stranded oligo DNAs were annealed and inserted into the BbsI site of px459. Then, these plasmids were transfected by Lipofectamine 3000 (Invitrogen, CA, USA) into cells at subconfluency. After 24 h, the transfected cells were treated with 1 μg/mL puromycin for selection of stably transfected cells. Knockout of the *SPINT1* or *SPINT2* gene was confirmed by genome sequencing, immunoblot analysis and immunocytochemistry. Immunocytochemistry was performed as described previously^7^. After incubation with primary antibody for 1 h and phosphate-buffered saline (PBS) washing, the cells were incubated for 30 min with Alexa Fluor 488-conjugated goat anti-mouse IgG (Life Technologies, Tokyo, Japan). Then, the cells were washed with PBS and counterstained with 2-(4-amidinophenyl)-1H-indole-6-carboxamidine (DAPI) (Life Technologies).

For reversion of defective HAI-2, the *SPINT2^−/−^* SAS cells were transfected with a HAI-2 expression vector, pCI-HAI-2. Briefly, the whole *SPINT2* coding region with three bases (GCC) preceding the initiation codon was inserted into the XbaI/SalI site of the pCIneo mammalian cell expression plasmid (Promega, Madison, WI, USA) generating the pCI-HAI-2. The plasmid was transfected into *SPINT2^−/−^* cells using Lipofectamine 3000 reagent (Invitrogen). After transfection, the cells were cultured in the presence of 0.5 mg/mL of G418 (Sigma), and a G418-resistant cell pool was obtained.

### Evaluation of cellular proliferation, migration and invasion *in vitro*

For determination of cellular proliferative capability *in vitro*, growth curve and colony-forming efficiencies were determined. To establish growth curves, triplicate 35-mm dishes were seeded at 3.0 × 10^4^ cells/2 mL growth medium. The number of viable cells was counted daily. To determine colony-forming efficiency, cells were seeded at 100 cells/2 mL in 35-mm dishes and cultured for ten days. The cell colonies were stained with crystal violet, and the number of visible colonies was counted. The colony-forming efficiency was then calculated as colony numbers formed per cells plated × 100. In an indicated experiment, cell number assessment by cell counting kit-8 (Wako, Osaka, Japan) was also performed according to the manufacturer’s instructions. For the evaluation of anchorage-independent growth, a soft agar colony formation assay was performed. Briefly, SAS cells (1.0×10^4^/well in 6-well plates) were cultured in a cell agar layer containing 0.36% agar (Nakarai, Kyoto, Japan) in growth medium placed on a base agar layer (0.75% agar in growth medium). The plates were incubated at 37°C for one week, and their colonies were observed under a phase-contrast microscope.

For evaluation of cellular migratory activity, a wound healing assay was performed. Briefly, cells were seeded in 6-well plates in growth medium. After the cells had formed a confluent monolayer, the layers were scratched using a sterile 1000-μL pipette tip and washed in PBS to remove cell debris. After a PBS wash, the cells were incubated with serum-free DMEM. The scratch wounds were photographed at 12 h with a ×4 objective. For evaluation of the invasive capability, Chemotaxicells (Greiner Bio-One, Kremsmünster, Austria) were coated with 25 μg of Matrigel (Life Sciences, Tewksbury, MA, USA) per filter and 5 × 10^5^ cells in DMEM with 0.1% bovine serum albumin (BSA) were seeded in the upper chamber and incubated for 48 h. The lower chamber contained medium with 5% FBS as a chemoattractant. The invading cells that had migrated to the bottom of the insert membrane were fixed and stained with hematoxylin.

### Immunoblot analysis

Cells at ~80% confluence were maintained in serum-free DMEM with ITS for 24 h under either normoxic or hypoxic condition as described. Then, serum-free conditioned media (SFCM) were collected and centrifuged at (185 × *g*) for 5 min to remove insoluble cellular debris, followed by 10-fold concentration by ultrafiltration using Amicon Ultra filter units (10kDa cut-off) (Millipore, Bedford, MA, USA). To obtain total cell lysates, cells were extracted with 1% Triton X-100 with protease inhibitor cocktail (1:40; Sigma). Cellular debris was removed by centrifugation (15400 × *g* for 15 min at 4°C). Protein concentration was measured with BCA protein assay kit (Thermo-Fischer). Equal amounts of proteins were subjected to standard sodium dodecyl sulfate (SDS)-polyacrylamide gel electrophoresis under reducing or non-reducing conditions and transferred onto an Immobilon™ membrane (Millipore). After blocking with 5% skim milk in 50 mM Tris-HCl, pH7.6, 138 mM NaCl, 2.7 mM KCl with 0.1% Tween 20 (TBS-T), the membrane was incubated with primary antibody overnight at 4°C, followed by washing in TBS-T and incubation with a peroxidase-conjugated goat anti-mouse IgG (Bio-Rad) or rabbit anti-goat IgG (DAKO, Glostrup, Denmark) diluted in TBS-T with 1% BSA for 1 h at room temperature. The labeled proteins were visualized with chemiluminescence reagent (PerkinElmer Life Science, Boston, MA, USA). For the analysis of N-glycosylation level of HAI-2, cellular extracts were treated with 25 μunits/μL peptide N-glycosidase F (Takara Bio, Shiga, Japan) in the presence of 1% SDS for 1 h at 37°C and the reactants were subjected to immunoblot analysis.

### RT-PCR and qRT-PCR

Total RNAs were prepared with TRIzol™ (Life Technologies), followed by DNAse I (Roche Applied Science, Indianapolis, IN, USA) treatment. For RT-PCR, 3μg total RNA was reverse transcribed with a mixture of oligo(dT)12-8 (Life Technologies) and random primers (TOYOBO, Osaka, Japan) using 200 units of ReverTra Ace™ (TOYOBO), and 1/30 of the resulting cDNA was processed for each PCR reaction and analyzed by 1.0% agarose gel electrophoresis as described previously [8]. Glyceraldehyde-3-phosphate dehydrogenase (GAPDH) and β-actin mRNAs were used as internal controls. For qRT-PCR, PCR was performed in a Thermal Cycler Dice Real Time System II (Takara Bio) using the SYBR Premix Ex Taq ll (Takara Bio), and the data were normalized to the β-actin mRNA level. All sequences of PCR primers are listed in Supplementary Table 1.

### Prostasin silencing and gelatin zymography

To knock down the prostasin (*PRSS8*) gene by small interfering RNA (siRNA), two kinds of Stealth™ siRNA (Invitrogen) were used. The sequences of the siRNAs were 5′-GGCCAUUCUGCUCUAUCUUGG AUUA-3′ (prostasin siRNA #1) and 5′-CAGGG CUUGCUGAGGCCCAUCCUUU-3′ (prostasin siRNA #2). Stealth siRNA Negative Control Duplexes (Invitrogen) were transfected as a control. Transfection was performed using Lipofectamine 3000 (Invitrogen).

For gelatin zymography, samples were electrophoresed in 10% SDS-polyacrylamide gels impregnated with 1 mg/mL gelatin (KOKEN, Tokyo, Japan) under non-reducing conditions. After electrophoresis, gels were washed in 50 mM Tris-HCl (pH 7.5) containing 0.1 M NaCl and 2.5% Triton-X-100 for 1.5 h to remove SDS, followed by incubation in 50 mM Tris-HCl at pH 7.5 with or without 0.5 mM EDTA and 10 μM broad-spectrum MMP inhibitor, GM6001 (Millipore), for 30 min to eliminate MMP activities. Then the gels were incubated at 37°C for 24 h in 50 mM Tris-HCl (pH 7.5) and stained with Coomassie Brilliant Blue.

### Xenotransplantation in nude mice

All animal procedures were performed in accordance with institutional guidelines, and the protocol was approved by the Animal Care Committee of the University of Miyazaki. SAS cells (1.0 × 10^6^) in 100 μL PBS were injected subcutaneously into the abdominal flanks of 6-week-old male nude mice (BALB/cAJc1-nu) without or with 1.0 × 10^6^ MRC5 fibroblasts. The tumor volume (mm^3^) was calculated by the formula: length x width x width/2. The mice were sacrificed and autopsied three weeks after the injection, and all of the xenograft tumors were excised and fixed with 4% formaldehyde in PBS. Paraffin embedded tissue sections were prepared from the maximum cut surface of each tumor.

### Immunohistochemical analysis of HAI-2 in human OSCC tissues

The study protocol was in accordance with the revised Helsinki Declaration of 1983 and approved by the Institutional Review Board of the Faculty of Medicine, University of Miyazaki (approved number: 2016-283). Formalin-fixed, paraffin-embedded tissue sections were prepared from surgical specimens of OSCC patients resected at the University of Miyazaki Hospital, Miyazaki, Japan. All specimens were obtained with informed consent from all patients. A total of 25 cases, none of whom had adjuvant therapy before surgery, were evaluated. After heat-induced epitope retrieval, the tissue sections were stained for HAI-2 using mAb XY-9 [18]. All procedures were performed using a Ventana automated staining system with a biotin-streptavidin system DAB Map kit and Amplification kit (Ventana Medical system, Tucson, AZ). For a negative control, a tissue section from nude mouse tumors from *SPINT2^−/−^* SAS was used. The omission of primary antibody was also used as another negative control. Immunoreactivity of minor salivary gland epithelium was used for the internal positive control.

To evaluate the HAI-2 expression level, we scored the areas of positive immunoreactivity similar to or stronger than internal control (minor salivary gland epithelium) and the score was graded as follows: 0, no positive area; 1, positive in <25% of area; 2, 25%≤ positive area <50%; 3, positive area ≥50%. We also scored the staining intensity as follows: 0, similar to or weaker than internal control; 1, stronger than internal control. The final immunoreactivity score (0 to 4) was a total of the area score and intensity score. Then the cases were sub-grouped into HAI-2-low (≤ final score 2) and HAI-2-high (final score ≥ 3) cases.

### TCGA data collection and analysis

HAI-2 (*SPINT2*) RNA-Seq expression data, available for OSCC, were retrieved from TCGA Research Network (http://cancergenome.nih.gov/). Data were extracted from the TCGA Head and Neck Squamous Cell Carcinoma Provisional study through the cBioPortal for Cancer Genomics website (http://www.cbioportal.org/) on the 15th of November, 2016. In total, we selected 377 OSCC patients with complete data, including patient viability that could be used for survival analysis.

### Statistical analysis

Comparison between two groups was performed with the Mann-Whitney U test or Student’s t-test or analysis of variance (ANOVA) with Fisher’s protected least significant difference (PLSD) test. Fisher’s exact test was used for assessment of the relationship between valuables. OS was estimated using the Kaplan-Meier method where groups were compared using the log-rank test. Data analysis was done using the StatView 5.0 program (SAS, Cary, NC, USA). Significance was set at *p* < 0.05.

## SUPPLEMENTARY MATERIALS FIGURES AND TABLES



## References

[1] Kataoka H, Miyata S, Uchinokura S, Itoh H (2003). Roles of hepatocyte growth factor (HGF) activator and HGF activator inhibitor in the pericellular activation of HGF/scatter factor. Cancer Metastasis Rev.

[2] Kawaguchi M, Kataoka H (2014). Mechanisms of hepatocyte growth factor activation in cancer tissues. Cancers (Basel).

[3] Tanabe LM, List K (2017). The Role of Type II transmembrane serine protease mediated signaling in cancer. FEBS J.

[4] Szabo R, Lantsman T, Peters DE, Bugge TH (2016). Delineation of proteolytic and non-proteolytic functions of membrane-anchored serine protease Prss8/prostasin. Development.

[5] Antalis TM, Buzza MS, Hodge KM, Hooper JD, Netzel-Arnett S (2010). The cutting edge: membrane-anchored serine protease activities in the pericellular microenvironment. Biochem J.

[6] Nagaike K, Kawaguchi M, Takeda N, Fukushima T, Sawaguchi A, Kohama K, Setoyama M, Kataoka H (2008). Defect of hepatocyte growth factor activator inhibitor type 1/serine protease inhibitor, Kunitz type 1 (Hai-1/Spint1) leads to ichthyosis-like condition and abnormal hair development in mice. Am J Pathol.

[7] Kawaguchi M, Kanemaru A, Yamamoto K, Baba T, Lin CY, Johnson MD, Fukushima T, Kataoka H (2015). Hepatocyte growth factor activator inhibitor type 1 maintains the assembly of keratin into desmosomes in keratinocytes by regulating protease-activated receptor 2-dependent p38 signaling. Am J Pathol.

[8] Baba T, Kawaguchi M, Fukushima T, Sato Y, Orikawa H, Yorita K, Tanaka H, Lin CY, Sakoda S, Kataoka H (2012). Loss of membrane-bound serine protease inhibitor HAI-1 induces oral squamous cell carcinoma cells’ invasiveness. J Pathol.

[9] Kanemaru A, Yamamoto K, Kawaguchi M, Fukushima T, Lin CY, Johnson MD, Camerer E, Kataoka H (2017). Deregulated matriptase activity in oral squamous cell carcinoma promotes the infiltration of cancer-associated fibroblasts by paracrine activation of protease-activated receptor 2. Int J Cancer.

[10] Cheng MF, Huang MS, Lin CS, Lin LH, Lee HS, Jiang JC, Hsia KT (2014). Expression of matriptase correlates with tumour progression and clinical prognosis in oral squamous cell carcinoma. Histopathology.

[11] List K, Szabo R, Molinolo A, Sriuranpong V, Redeye V, Murdock T, Burke B, Nielsen BS, Gutkind JS, Bugge TH (2005). Deregulated matriptase causes ras-independent multistage carcinogenesis and promotes ras-mediated malignant transformation. Genes Dev.

[12] Szabo R, Rasmussen AL, Moyer AB, Kosa P, Schafer JM, Molinolo AA, Gutkind JS, Bugge TH (2011). c-Met-induced epithelial carcinogenesis is initiated by the serine protease matriptase. Oncogene.

[13] Sales KU, Friis S, Abusleme L, Moutsopoulos NM, Bugge TH (2015). Matriptase promotes inflammatory cell accumulation and progression of established epidermal tumors. Oncogene.

[14] Szabo R, Hobson JP, List K, Molinolo A, Lin CY, Bugge TH (2008). Potent inhibition and global co-localization implicate the transmembrane Kunitz-type serine protease inhibitor hepatocyte growth factor activator inhibitor-2 in the regulation of epithelial matriptase activity. J Biol Chem.

[15] Chang HH, Xu Y, Lai H, Yang X, Tseng CC, Lai YJ, Pan Y, Zhou E, Johnson MD, Wang JK, Lin CY (2015). Differential subcellular localization renders HAI-2 a matriptase inhibitor in breast cancer cells but not in mammary epithelial cells. PLoS One.

[16] Jemal A, Bray F, Ferlay J (2011). Global cancer statistics. CA Cancer J Clin.

[17] Sinevici N, O’Sullivan J (2016). Oral cancer: Deregulated molecular events and their use as biomarkers. Oral Oncol.

[18] Lai YJ, Chang HH, Lai H, Xu Y, Shiao F, Huang N, Li L, Lee MS, Johnson MD, Wang JK, Lin CY (2015). N-Glycan branching affects the subcellular distribution of and inhibition of matriptase by HAI-2/Placental Bikunin. PLoS One.

[19] Cheng H, Fukushima T, Takahashi N, Tanaka H, Kataoka H (2009). Hepatocyte growth factor activator inhibitor type 1 regulates epithelial to mesenchymal transition through membrane-bound serine proteinases. Cancer Res.

[20] Fukushima T, Kawaguchi M, Yamasaki M, Tanaka H, Yorita K, Kataoka H (2011). Hepatocyte growth factor activator inhibitor type 1 suppresses metastatic pulmonary colonization of pancreatic carcinoma cells. Cancer Sci.

[21] Ye J, Kawaguchi M, Haruyama Y, Kanemaru A, Fukushima T, Yamamoto K, Lin CY, Kataoka H (2014). Loss of hepatocyte growth factor activator inhibitor type 1 participates in metastatic spreading of human pancreatic cancer cells in a mouse orthotopic transplantation model. Cancer Sci.

[22] Friis S, Sales KU, Schafer JM, Vogel LK, Kataoka H, Bugge TH (2014). The Protease Inhibitor HAI-2, but Not HAI-1, Regulates Matriptase Activation and Shedding through Prostasin. J Biol Chem.

[23] Lai CH, Lai YJ, Chou FP, Chang HH, Tseng CC, Johnson MD, Wang JK, Lin CY (2016). Matriptase complexs with HAI-1 and HAI-2 in human milk: significant proteolysis in lactation. PLoS One.

[24] Antalis TM, Bugge TH, Wu Q (2011). Membrane-anchored serine proteases in health and disease. Prog Mol Biol Transl Sci.

[25] Friis S, Sales KU, Godiksen S, Peters DE, Lin CY, Vogel LK, Bugge TH (2013). A matriptase-prostasin reciprocal zymogen activation complex with unique features: Prostasin as a non-enzymatic co-factor for matriptase activation. J Biol Chem.

[26] Su HC, Liang YA, Lai YJ, Chiu YL, Barndt RB, Shiao F, Chang HD, Lu DD, Huang N, Tseng CC, Wang JK, Lee MS, Johnson MD (2016). Natural Endogenous Human Matriptase and Prostasin Undergo Zymogen Activation via Independent Mechanisms in an Uncoupled Manner. PLoS One.

[27] Kataoka H, Itoh H, Uchino H, Hamasuna R, Kitamura N, Nabeshima K, Koono M (2000). Conversed expression of hepatocyte growth factor activator inhibitor type-2/placental bikunin in human colorectal carcinoma. Cancer Lett.

[28] Larsen BR, Steffensen SD, Nielsen NV, Friis S, Godiksen S, Bornholdt J, Soendergaard C, Nonboe AW, Andersen MN, Poulsen SS, Szabo R, Bugge TH, Lin CY (2013). Hepatocyte growth factor activator inhibitor-2 prevents shedding of matriptase. Exp Cell Res.

[29] Muller-Pillasch F, Wallrapp C, Bartels K, Varga G, Friess H, Buchler M, Adler G, Gress TM (1998). Cloning of a new Kunitz-type protease inhibitor with a putative transmembrane domain overexpressed in pancreatic cancer. Biochim Biophys Acta.

[30] Fukai K, Yokosuka O, Chiba T, Hirasawa Y, Tada M, Imazeki F, Kataoka H, Saisho H (2003). Hepatocyte growth factor activator inhibitor 2/placental bikunin (HAI-2/PB) gene is frequently hypermethylated in human hepatocellular carcinoma. Cancer Res.

[31] Morris MR, Gentle D, Abdulrahman M, Clarke N, Brown M, Kishida T, Yao M, Teh BT, Latif F, Maher ER (2008). Functional epigenomics approach to identify methylated candidate tumour suppressor genes in renal cell carcinoma. Br J Cancer.

[32] Hwang S, Kim HE, Min M, Raghunathan R, Panova IP, Munshi R, Ryu B (2015). Epigenetic silencing of SPINT2 promotes cancer cell motility via HGF-MET pathway activation in melanoma. J Invest Dermatol.

[33] Bode W, Maskos K (2011). Matrix Metalloproteinases. Encycl Inorg Bioinorg Chem.

[34] Eatemadi A, Aiyelabegan HT, Negahdari B, Mazlomi MA, Daraee H, Eatemadi R, Sadroddiny E (2017). Role of protease and protease inhibitors in cancer pathogenesis and treatment. Biomed Pharmacother.

[35] Heinz-Erian P, Müller T, Krabichler B, Schranz M, Becker C, Rüschendorf F, Nürnberg P, Rossier B, Vujic M, Booth IW, Holmberg C, Wijmenga C, Grigelioniene G (2008). Mutation in SPINT2 cause a Syndromic form of congenital sodium diarrhea. Am J Hum Genet.

[36] Shiao F, Liu LO, Huang N, Lai YJ, Barndt RJ, Tseng C, Wang JK, Jia B, Johnson MD, Lin CY (2017). Selective inhibition of prostasin in human enterocytes by the integral membrane Kunitz-type serine protease inhibitor HAI-2. PLoS One.

[37] Chen LM, Hodge GB, Guarda LA, Welch JL, Greenberg NM, Chai KX (2001). Down-regulation of prostasin serine protease: a potential invasion suppressor in prostate cancer. Prostate.

[38] Chen LM, Chai KX (2002). Prostasin serine protease inhibits breast cancer invasiveness and is transcriptionally regulated by promoter DNA methylation. Int J Cancer.

[39] Ma XJ, Fu YY, Li YX, Chen LM, Chai K, Wang YL (2009). Prostasin inhibits cell invasion in human choriocarcinomal JEG-3 cells. Histochem Cell Biol.

[40] Zhang L, Jia G, Shi B, Ge G, Duan H, Yang Y (2016). PRSS8 is downregulated and suppresses tumour growth and metastases in hepatocellular carcinoma. Cell Physiol Biochem.

[41] Chen LM, Verity NJ, Chai KX (2009). Loss of prostasin (PRSS8) in human bladder transitional cell carcinoma cell lines is associated with epithelial-mesenchymal transition (EMT). BMC Cancer.

[42] Bao Y, Wang Q, Guo Y, Chen Z, Li K, Yang Y, Zhang H, Dong H, Shen K, Yang W (2016). PRSS8 methylation and its significance in esophageal squamous cell carcinoma. Oncotarget.

[43] Takahashi S, Suzuki S, Inaguma S, Ikeda Y, Cho YM, Hayashi N, Inoue T, Sugimura Y, Nishiyama N, Fujita T, Chao J, Ushijima T, Shirai T (2003). Down-regulated expression of prostasin in high-grade or hormone-refractory human prostate cancers. Prostate.

[44] Chen PX, Li QY, Yang Z (2013). Axl and prostasin are biomarkers for prognosis of ovarian adenocarcinoma. Ann Diagn Pathol.

[45] Sakashita K, Mimori K, Tanaka F, Tahara K, Inoue H, Sawada T, Ohira M, Hirakawa K, Mori M (2008). Clinical significance of low expression of prostasin mRNA in human gastric cancer. J Surg Oncol.

[46] Chen M, Fu YY, Lin CY, Chen LM, Chai KX (2007). Prostasin induces protease-dependent and independent molecular changes in the human prostate carcinoma cell line PC-3. Biochim Biophys Acta.

[47] Hamasuna R, Kataoka H, Meng JY, Itoh H, Moriyama T, Wakisaka S, Koono M (2001). Reduced expression of hepatocyte growth factor activator inhibitor type-2/placental bikunin (HAI-2/PB) in human glioblastomas: Implication for anti-invasive role of HAI-2/PB in glioblastoma cells. Int J Cancer.

[48] Yamauchi M, Kataoka H, Itoh H, Seguchi T, Hasui Y, Osada Y (2004). Hepatocyte growth factor activator inhibitor types 1 and 2 are expressed by tubular epithelium in kidney and down-regulated in renal cell carcinoma. J Urol.

[49] Morris MR, Gentle D, Abdulrahman M, Mania EN, Gupta K, Banks RE, Wiesener MS, Kishida T, Yao M, Teh B, Latif F, Maher ER (2005). Tumor suppressor activity and epigenetic inactivation of hepatocyte growth factor activator inhibitor type 2/SPINT2 in papillary and clear cell renal cell carcinoma. Cancer Res.

[50] Kongkham PN, Northcott PA, Ra YS, Nakahara Y, Mainprize TG, Croul SE, Smith CA, Taylor MD, Rutka JT (2008). An epigenetic genome-wide screen identifies SPINT2 as a novel tumor suppressor gene in pediatric medulloblastoma. Cancer Res.

[51] Yue D, Fan Q, Chen X, Li F, Wang L, Huang L, Dong W, Chen X, Zhang Z, Liu J, Wang F, Wang M, Zhang B, Zang Y (2014). Epigenetic inactivation of SPINT2 is associated with tumor suppressive function in esophageal squamous cell carcinoma. Exp Cell Res.

[52] Dong W, Chen X, Xie J, Sun P, Wu Y (2010). Epigenetic inactivation and tumor suppressor activity of HAI-2/SPINT2 in gastric cancer. Int J Cancer.

[53] Bergum C, List K (2010). Loss of the matriptase inhibitor HAI-2 during prostate cancer progression. Prostate.

[54] Tsai CH, Teng CH, Tu YT, Cheng TS, Wu SR, Ko CJ, Shyu HY, Lan SW, Huang HP, Tzeng SF, Johnson MD, Lin CY, Hsiao PW, Lee MS (2014). HAI-2 suppresses the invasive growth and metastasis of prostate cancer through regulation of matriptase. Oncogene.

[55] Nakamura K, Abarzua F, Kodama J, Hongo A, Nasu Y, Kumon H, Hiramatsu Y (2009). Expression of hepatocyte growth factor activator inhibitors (HAI-1 and HAI-2) in ovarian cancer. Int J Oncol.

[56] Nakamura K, Abarzua F, Hongo A, Kodama J, Nasu Y, Kumon H, Hiramatsu Y (2009). Hepatocyte growth factor activator inhibitor-2 (HAI-2) is a favorable prognosis marker and inhibits cell growth through the apoptotic pathway in cervical cancer. Ann Oncol.

[57] Generali D, Fox SB, Berruti A, Moore JW, Brizzi MP, Patel N, Allevi G, Bonardi S, Aquqqini S, Bersiga A, Campo L, Dogliotti L, Bottini A, Harris AL (2007). Regulation of hepatocyte growth factor activator inhibitoe 2 by hypoxia in breast cancer. Clin Cancer Res.

[58] Lin CY, Anders J, Johnson M, Dickson RB (1999). Purification and characterization of a complex containing matriptase and a Kunitz-type serine protease inhibitor from human milk. J Biol Chem.

[59] Xu H, Xu Z, Tseng IC, Chou FP, Chen YW, Wang JK, Johnson MD, Kataoka H, Lin CY (2012). Mechanisms for the control of matriptase activity in the absence of sufficient HAI-1. Am J Physiol Cell Physiol.

[60] Kataoka H, Suganuma T, Shimomura T, Itoh H, Kitamura N, Nabeshima K, Koono M (1999). Distribution of hepatocyte growth factor activator inhibitor type 1 (HAI-1) in human tissues. Cellular surface localization of HAI-1 in simple columnar epithelium and its modulated expression in injured and regenerative tissues. Histochem Cytochem.

