# Supplementary material for: Hepatocyte growth factor activator inhibitor type-2 (HAI-2)/SPINT2 contributes to invasive growth of oral squamous cell carcinoma cells
Source: Oncotarget. 2018 Feb 8;9(14):11691–706. doi: 10.18632/oncotarget.24450 (PMC5837738; doi:10.18632/oncotarget.24450)
Supplement: Supplementary file 1 [file oncotarget-09-11691-s001.pdf]

# Hepatocyte growth factor activator inhibitor type-2 (HAI-2)/*SPINT2* contributes to invasive growth of oral squamous cell carcinoma cells

## SUPPLEMENTARY MATERIALS

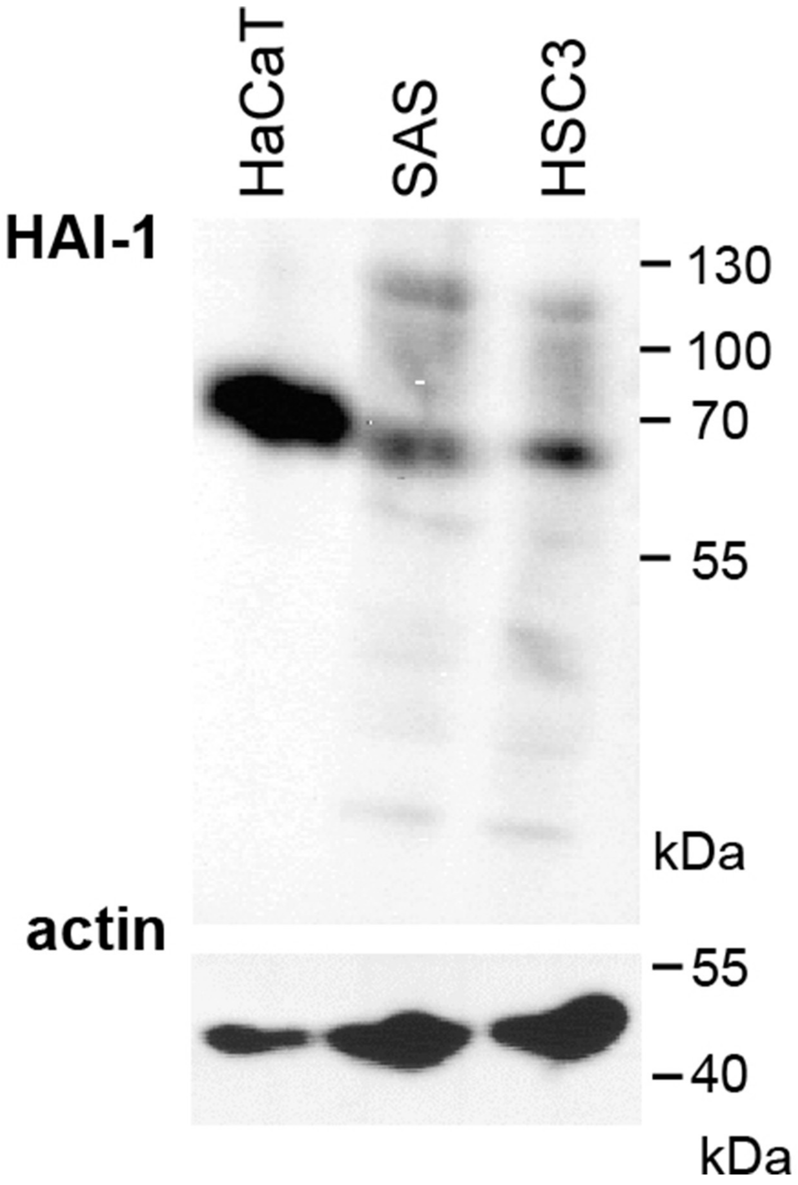


**Supplementary Figure 1: Immunoblot analysis of HAI-1 in cellular extracts.**

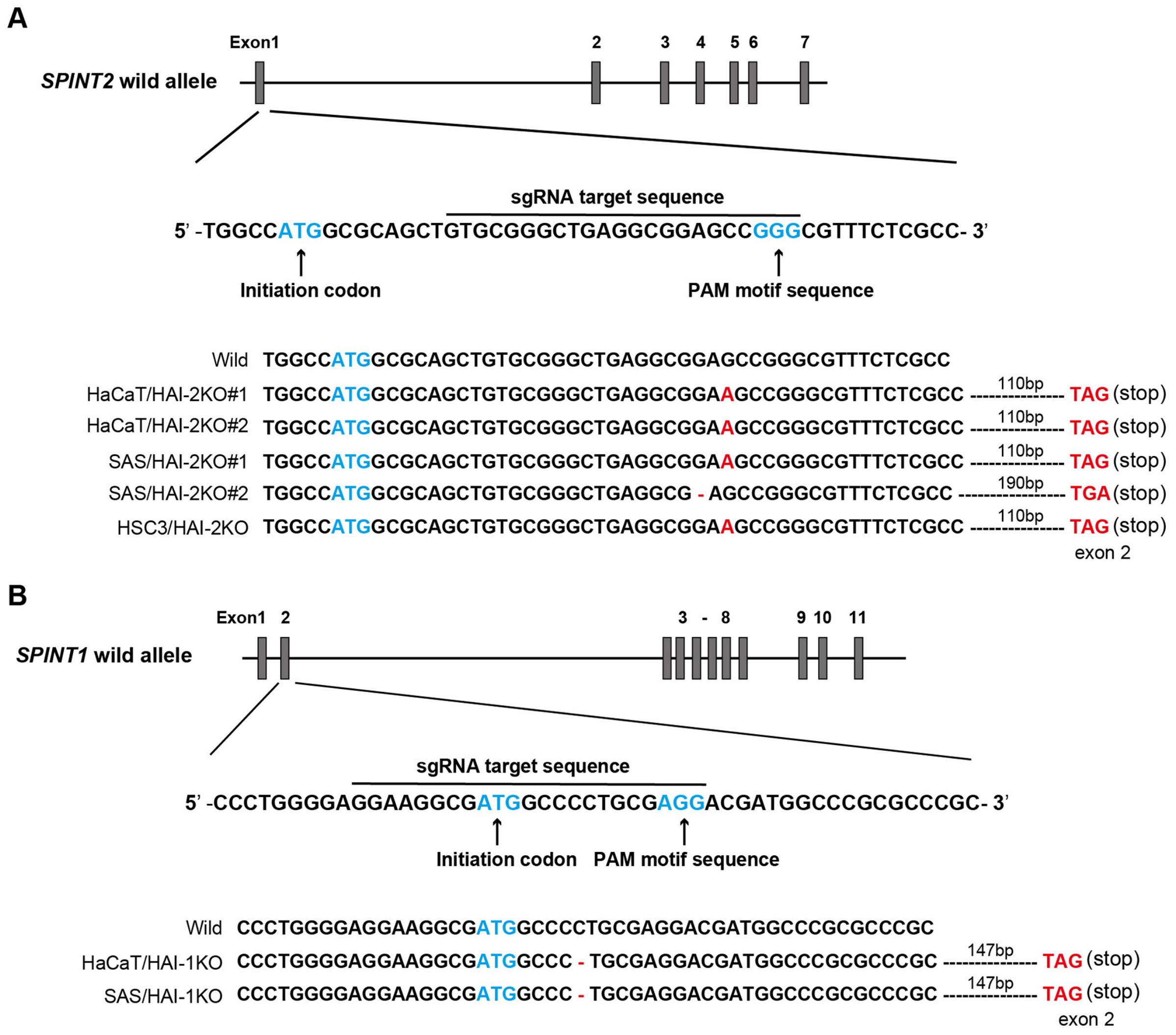


**Supplementary Figure 2:** Knockout of *SPINT2* **(A)** and *SPINT1* **(B)** by CRISPR/Cas9. Targeting strategy and sequences of mutant clones are indicated.

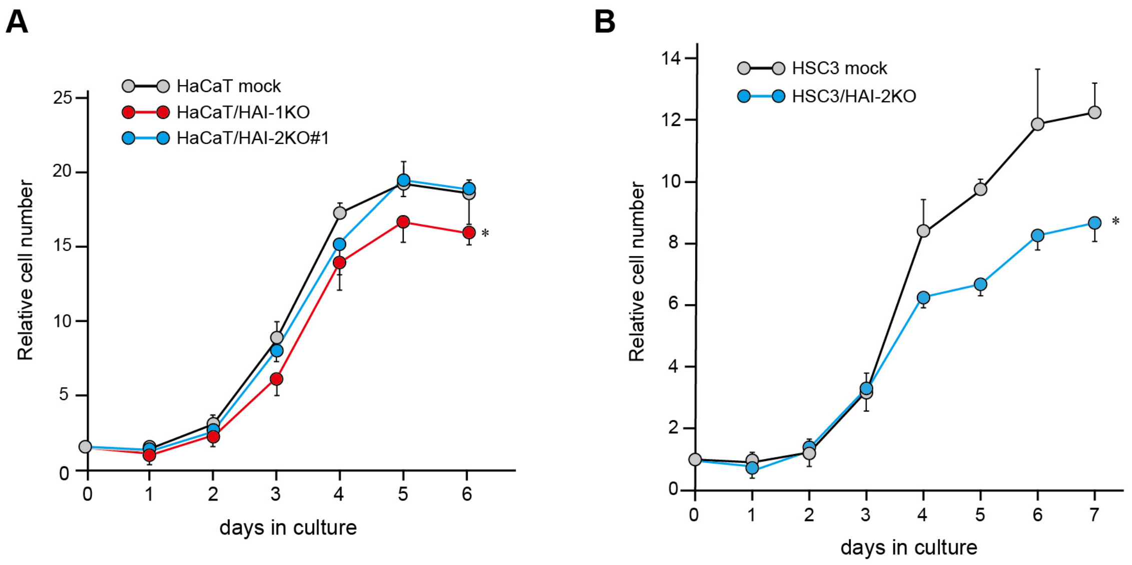


**Supplementary Figure 3: Growth curve of HaCaT and HSC3 and their mutants.** Cells were cultured in DMEM supplemented with 10% FBS under a normoxic condition. **(A)** HaCaT cells. $^{*}$, $p < 0.01$ compared to mock and HAI-2KO#1 (n = 3, ANOVA with Fisher's PLSD test). **(B)** HSC3 cells. $^{*}$, $p < 0.01$ compared to mock (n = 3, ANOVA with Fisher's PLSD test).

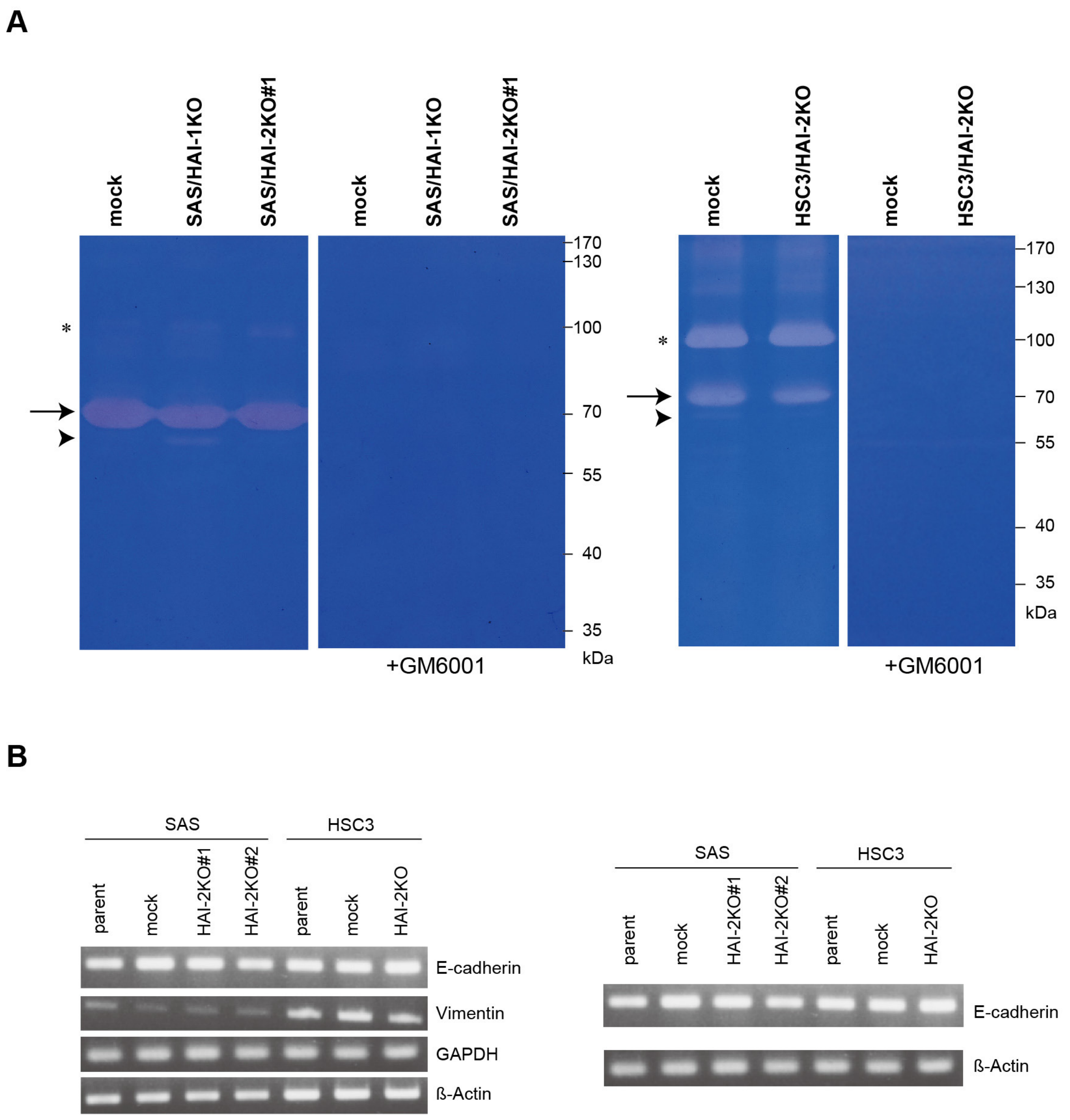


**Supplementary Figure 4: Analysis of gelatinolytic activity and expression of EMT-related molecules. (A)** Gelatin zymography of serum-free culture conditioned medium with or without MMP inhibitor GM6001. Arrow, arrowhead and asterisk indicate presumed MMP-2 proform, MMP-2 active form, and MMP-9, respectively. **(B)** Expression of E-cadherin and vimentin in control and HAI-2KO OSCC cells. Data of RT-PCR (left panel) and immunoblot (right panel) are shown.

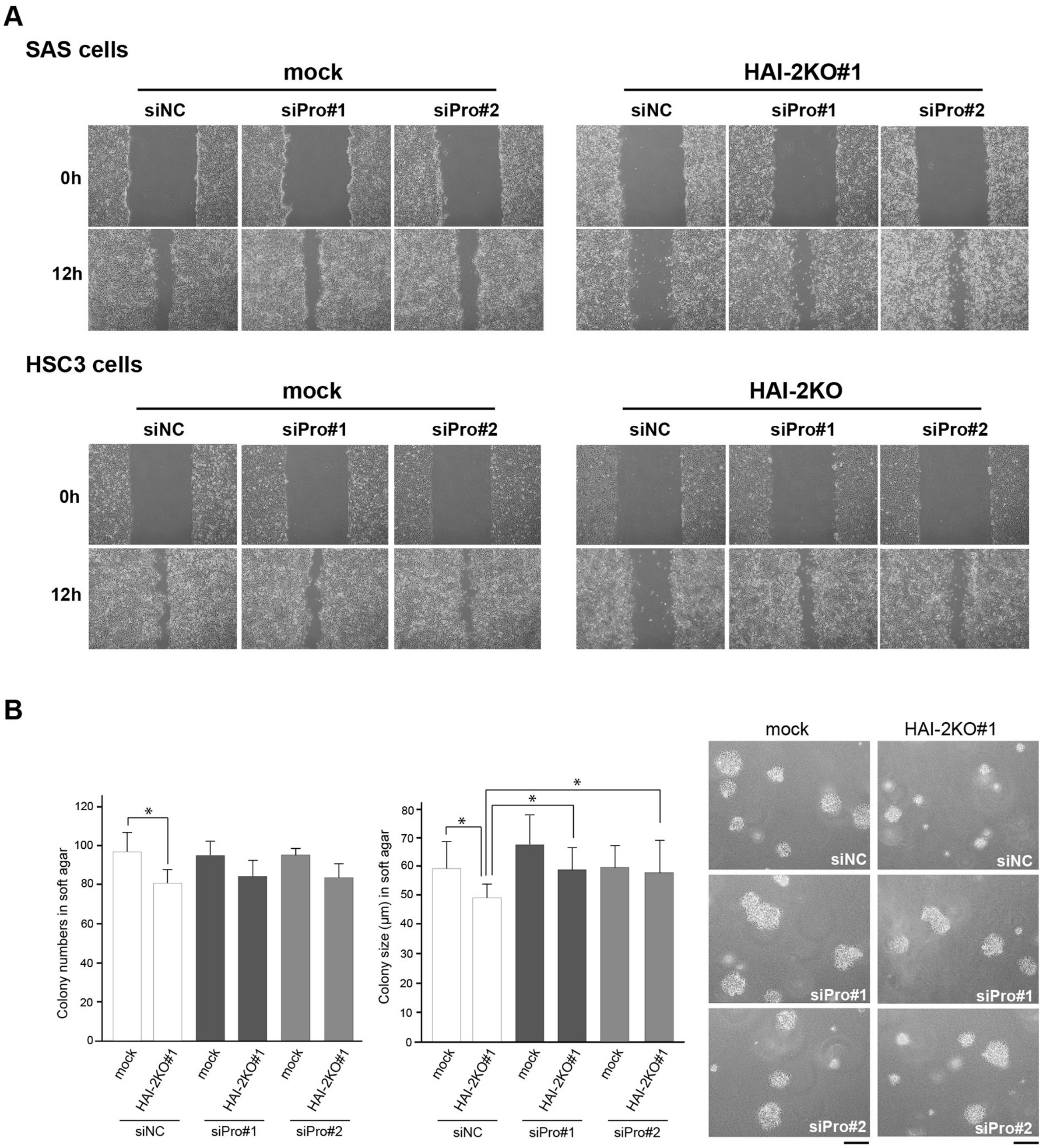


**Supplementary Figure 5: Effects of prostasin silencing on wound healing assay and anchorage-independent growth in soft agar. (A)** Photos of wound healing assays for each set experiments. **(B)** Means ± SD of colony number per ×40 field (left graph) and colony diameter (right graph, µm) of SAS cells in soft agar. N = 9 for each group; $^{*}$, $p < 0.01$ Mann-Whitney U test. Representative photos are also shown. Bar, 50 µm.

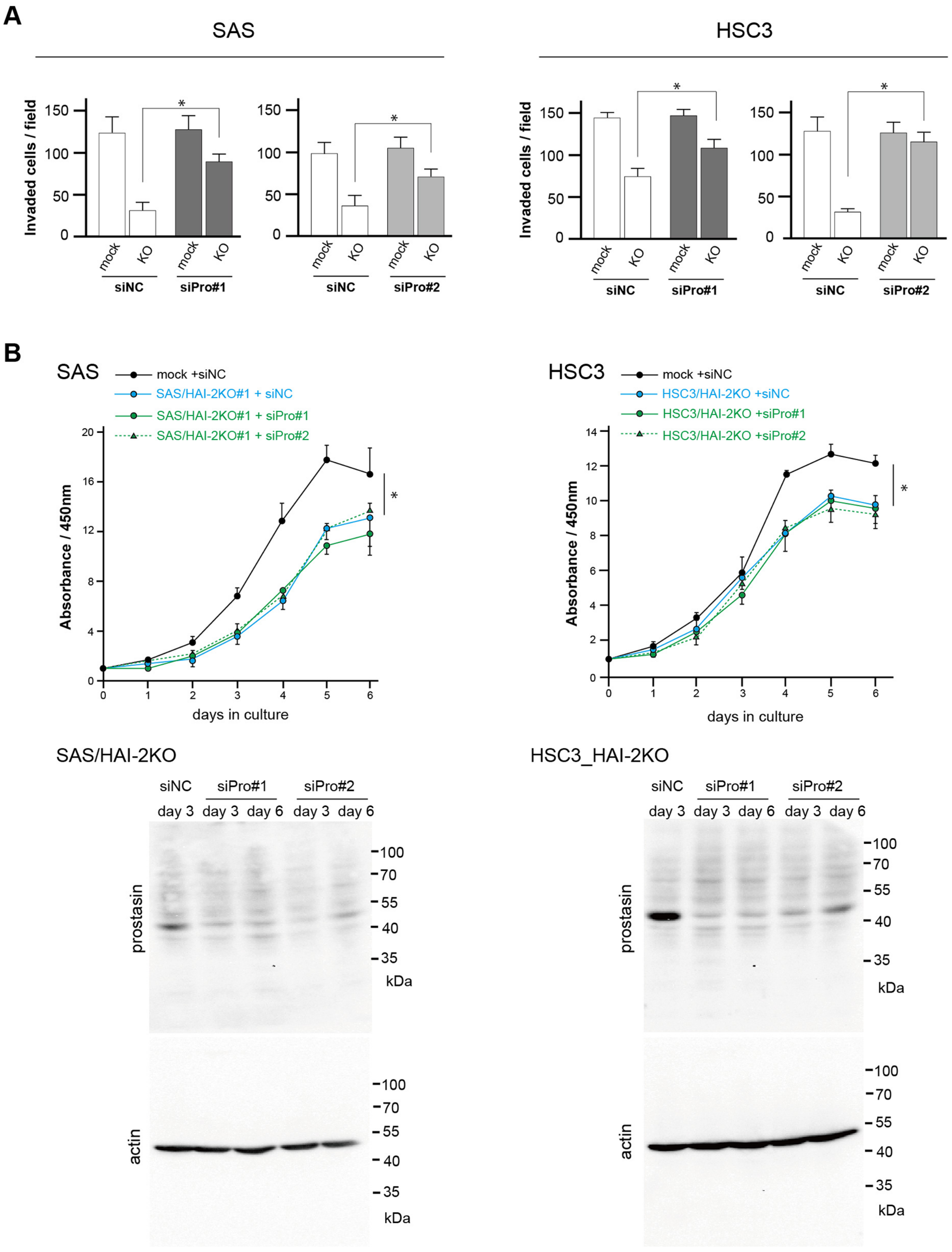


**Supplementary Figure 6: Effects of prostasin silencing on invasion under hypoxic condition and cellular growth rate on culture dishes under normoxic condition. (A)** Effect of prostasin silencing on Matrigel invasion under hypoxic condition. *, $p < 0.01$; n = 8, Mann-Whitney U test. **(B)** Effect of prostasin silencing on cellular proliferation under normoxic condition. The cell number was estimated by cell counting kit 8 (Wako, Osaka, Japan). 1000 cells were plated in 96-well culture plates. The effects of transiently transfected siRNA on prostasin levels were checked by immunoblot analysis of cellular extracts at 3 and 6 days after plating (lower panel). *, $p < 0.001$; n = 3, ANOVA with Fisher's PLSD.

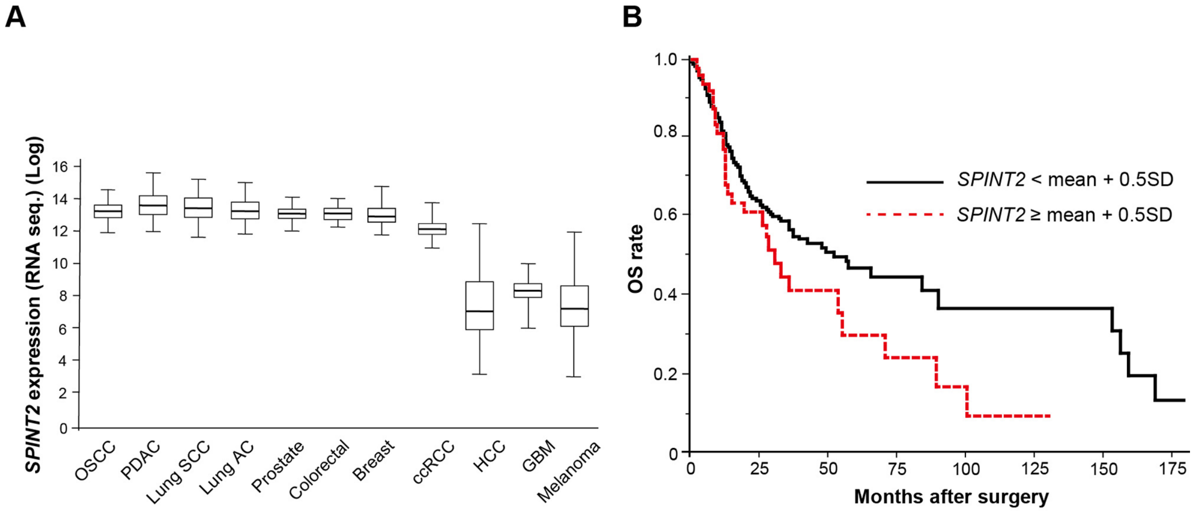


**Supplementary Figure 7: Expression of *SPINT2* mRNA in OSCC and its relationship to the survival of OSCC patients.** **(A)** *SPINT2* mRNA expression levels (RNA seq.) retrieved from TCGA. Data were extracted through the cBioPortal for Cancer Genomics website (http://www.cbioportal.org/). The box shows the interquartile range, the whiskers the largest and smallest observed scores, and the median is indicated by a bold vertical line. **(B)** Kaplan-Meier analysis of the RNA-Seq data and outcomes from 377 OSCC cases in the TCGA. Whereas high expression (≥ mean + 0.5SD; n = 49) of the *SPINT2* gene tended to be associated with decreased OS, the difference was not statistically significant ($p = 0.0682$; log-rank test).

**Supplementary Table 1: Primer sequences for RT-PCR**

| Target | Forward primer | Reverse primer | Size (bp) |
|---|---|---|---|
| Matriptase | 5′-CTTTGAGGCCACCTTCTTC-3′ | 5′-GGTAGTGGCCTGGGTAGTA-3′ | 104 |
| Hepsin | 5′-GTTCTCCTCAGGAGTGACCAGGA-3′ | 5′-AGAAGAAGCCCGACGTGCCATTGGC-3′ | 232 |
| TMPRSS2 | 5′-CCTGATCACACCAGCCATGATCTG-3′ | 5′-TTAGCCGTCTGCCCTCATTTGTCG-3′ | 226 |
| TMPRSS4 | 5′-ACCGATGTGTTCAACTGGAAG-3′ | 5′-CATCCAATGATCCAGAGTGG-3′ | 237 |
| TMPRSS13 | 5′-TGGTGGTTTCGCTCATCAT-3′ | 5′-ACAGCGAACAGCGTGCTT-3′ | 101 |
| Polyserase-1 | 5′-CGCTTCTACCCAGTGCAGATCA-3′ | 5′-GTGACCCCAGTTAGCACCCACC-3′ | 140 |
| Testisin | 5′-CTTAAGCTTATGGGCGCGCGCGGG-3′ | 5′-CAACTCGAGTTAGACCGGCCCCAGGAG-3′ | 926 |
| HAT | 5′-AGTTCCAGAGCTAAGGCAAGGACA-3′ | 5′-ATAAACCAAAGCCGCCGTGA-3′ | 189 |
| DESC1 | 5′-GGAATAGTGAGCTCGGGAGATG-3′ | 5′-TGCATCAAGCAAACAGTTTATTGAGATC-3′ | 243 |
| Corin | 5′-ACCTGTGGTGTGATGGTGAAGC-3′ | 5′-ATATCTCCTGCCAGCCATCTGC-3′ | 153 |
| Prostasin | 5′-TCTATCTTGGATTACTCCGGTCGG-3′ | 5′-ACATGGACGCCTTCATAGGTGATG-3′ | 151 |
| E-cadherin | 5′-ACACCATCCTCAGCCAAGATCC-3′ | 5′-GTGGTGGGATTGAAGATCGGAG-3′ | 232 |
| Vimentin | 5′- AGGAAATGGCTCGTCA CCTTCGTGAATA -3′ | 5′-AGGAGTTCGGTTGTTA AGAACTAGAGC-3′ | 440 |
| β-actin | 5′-ATTGCCGACAGGATGCAGA-3′ | 5′-GAGTACTTGCGCTCAGGAGGA-3′ | 89 |
| GAPDH | 5′-GTGAAGGTCGGAGTCAACG-3′ | 5′-GGTGAAGACGCCAGTGGACTC-3′ | 300 |
